# Synthesis and elucidation of strained galactopyronose esters as selective cyclooxygenase-2 inhibitor: a comprehensive computational approach†

**DOI:** 10.1039/d4ra03520h

**Published:** 2024-09-24

**Authors:** Mohammed Sakib Musa, Md. Sopon Miah, Yeasmin Akter Munni, Md. Abdul Majed Patwary, Mohsin Kazi, Mohammed Mahbubul Matin

**Affiliations:** a Department of Applied Chemistry and Chemical Engineering, Faculty of Science, University of Chittagong Chittagong 4331 Bangladesh; b Bioorganic and Medicinal Chemistry Laboratory, Department of Chemistry, Faculty of Science, University of Chittagong Chittagong 4331 Bangladesh mahbubchem@cu.ac.bd +880 1716 839689; c Department of Anatomy, College of Medicine, Dongguk University Gyeongju 38066 Republic of Korea; d Department of Chemistry, Comilla University Cumilla 3506 Bangladesh; e Department of Pharmaceutics, College of Pharmacy, King Saud University P. O. Box 2457 Riyadh 11451 Saudi Arabia mkazi@ksu.edu.sa

## Abstract

Cyclooxygenase-2 (COX-2) is critically implicated in various pathologies, including inflammation, cancer, disorders involving the nervous system, and multidrug resistance. In both academic and pharmaceutical research, the development of COX-2 selective drugs as anti-inflammatory and anti-tumor therapeutics is a key focus. Traditional nonsteroidal anti-inflammatory drugs (NSAIDs) have ulcerogenic, gastrointestinal adverse effects, and myocardial infarction risk, which resulted in their limited applications. In response to this challenge, we synthesized a series of glycoconjugates featuring six-membered sugar rings and acyl chains of varying lengths attached at the C-6 position. Using molecular docking techniques, we identified galactose esters with optimal acyl chain lengths that selectively and effectively bind to the active site of COX-2 over COX-1. These compounds exhibited enhanced binding affinity and superior inhibition constants (p*K*_i_) for COX-2, thereby offering selective inhibition with potentially reduced ulcerogenic risks, as COX-1 inhibition is thought to contribute to these side effects. The molecular docking study identified two potential compounds, G6 and G8, which were validated *via* MD simulation for the assessment of their stability and were compared to the complex of the standard drugs, aspirin and rofecoxib. In addition, compound structures were optimized using the DFT method under the B3LYP/6-31+g(d,p) basis set to study their physio-spectral properties, frontier molecular orbitals (HOMO–LUMO), and their energy gap that correlates to their reactivity and stability. ADMET, drug-likeness, and PASS analyses were also carried out to assess their drug-ability and toxicity profiling.

## Abstract 2

A novel class of SEs-based NSAIDs is produced by the synthesis and selective inhibition of cyclooxygenase-2 (COX-2) by strained acyl galactopyranoses.

## Graphical abstract

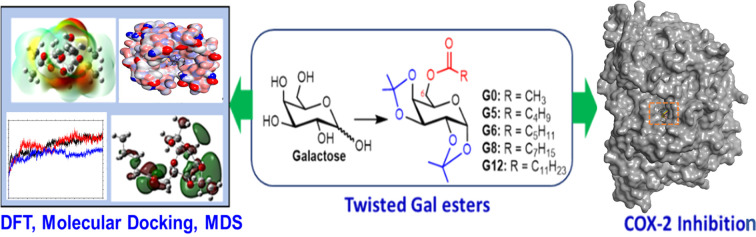

## 1 Introduction

Natural occurring carbohydrates, particularly those with monosaccharide structures, have a distinctive and large structural diversity that makes them the ideal identification markers for a variety of physiological functions.^1^ Compared to proteins and lipids, carbohydrates are shown to have a far higher structural variety, which may be due to the unique biological actions they have.^2^ Galactose (Gal), a C-4 epimer of d-glucose, is one of the carbohydrates that have been extensively employed to create biologically useful glycoconjugates in living things.^3^ In addition to its nutritional and metabolic benefits, as some prior research revealed, monosaccharides (*e.g.* galactose) offer a number of medicinal purposes.^4–8^ For instance, it has been discovered that galactose plays a crucial role in the binding of a variety of viruses, selectins, and toxins.^9^

Like many other naturally occurring carbohydrates, parent galactose has low binding affinities, which has led to structural modifications that have increased its effectiveness in a range of settings, including potential medicinal applications.^10^ Sugar esters (SEs), with a variety of uses, were synthesized by structural alteration of monosaccharide sugar with one or more acyl group(s).^11,12^ According to some studies, galactose conjugates showed involvement in targeting hepatocytes responsible for tumor cells.^13^ Additionally, SEs may have efficacy against the primary protease of SARS-CoV-2.^14^ Acyl galactopyranosides, like 5-aminosalicylic acid ester, have also been discovered to have a higher level of activity against *S. aureus* and *E. coli* in addition to being effective in treating inflammatory bowel disease (IBD).^15^ SEs have both hydrophilic and lipophilic moieties.^16^ By regulating their hydrophobic alkyl chains, these synthetic SE-protected derivatives have been found to be ideal for boosting therapeutic attributes in hyperproliferative and inflammatory agents and opening the path to the preparation of novel bioactive compounds.^17^ All these aforementioned therapeutic potentials of SEs and a prior *in vivo* investigation of glucose–aspirin conjugate as an anti-inflammatory and analgesic agent^18^ encouraged us to synthesize galactopyranose esters and evaluate their potency as an anti-inflammatory agent.

Non-steroidal anti-inflammatory medicines (NSAIDs) are among the most widely prescribed drugs worldwide. These medications are frequently applied to treat fever, pain, and inflammation in conditions like rheumatoid arthritis (RA) and osteoarthritis (OA). NSAIDs are structurally diverse, yet they all inhibit COX. The COX pathway produces prostaglandins (PGs), the byproducts of fatty acid (arachidonic acid) metabolism. In a variety of therapeutic areas, such as inflammation, pain, pyrexia, cancer, and neurological illnesses, PGs have long been recognized to serve as important physiological and pathological mediators.^19^ There are three isoforms of cyclooxygenase known as COX-1, COX-2, and COX-3. However, the main focus of attention is on COX-1 and COX-2 isoforms, since they are engaged in both physiological and pathological activities. Most tissues express COX-1 constitutively, which enables this isozyme to generate PGs that support homeostasis, heart functioning, and stomach mucosa lining protection. On the other hand, COX-2 is only constitutively expressed in a few organs, most notably the kidney and brain, but it is quickly activated by inflammatory mediators like IL-1 and LPS. According to,^20^ inflammatory regions have higher levels of COX-2 expression.

Evidence exists to support the crucial involvement of COX-2 in a number of pathologies, like inflammation,^21^ cancer,^22–24^ neurological illnesses,^25^ and multidrug resistance.^26^ Thus, one of the main areas of focus for both academic research and the pharmaceutical sector is particularly COX-2 inhibitors' development as anti-inflammatory and anti-tumor medications. The use of traditional NSAIDs (aspirin, ibuprofen, and naproxen) is limited because of their gastrointestinal and ulcerogenic side effects. Since they inhibit both COX-1 and COX-2, suppression of housekeeping COX-1 is a plausible cause of their adverse side effects. Again, a study suggests chronic use of selective COX-2 inhibitors (COXIBs) may lead to myocardial infarction.^27^ Developing medications that preferentially inhibit COX-2 over COX-1 can be difficult since both isoforms have very similar molecular weights, sites of cellular expression, and amino acid compositions. Furthermore, the two isoforms have almost identical three-dimensional structures and about 60% sequence homology. However, at positions 434 and 523, isoleucine in COX-1 is replaced by valine in COX-2, which is the primary distinction between the COX-1 and COX-2 active sites. The COX-2's substrate-binding site is about 25% bigger (394 Å^3^ in COX-2, 316 Å^3^ in COX-1) and more flexible due to the change in amino acid sequence, which also creates a unique secondary-binding pocket.^28^ Our synthesized galactose esters contain a bulkier acyl chain than that of aspirin, and the active pocket of COX-2 can accommodate these ligands more accurately due to its higher volume. In this study, the structural differences between the two isoenzymes guided our decision to prioritize COX-2 over COX-1, specifically targeting its active site for the synthesized SEs. This provided the basis for proposing the compounds as selective COX-2 inhibitors.

This work is distinguished by the innovative incorporation of galactopyranose-derived esters as a new chemical scaffold, which has not been previously investigated in this context. Initial molecular docking studies, supported by subsequent QSAR modeling, have revealed this scaffold's promising selectivity for COX-2 over COX-1. This novel structural framework not only expands the chemical space available for the development of COX-2 inhibitors but also lays the groundwork for future efforts aimed at optimizing and enhancing the selectivity and potency of these inhibitors.

## 2 Materials and methods

### 2.1. Synthetic methods

The reagents employed for the synthesis were purchased from suppliers (Aldrich and Merck) and used directly without further purification. The necessary solvents have been purified and dried by the usual methods. Kieselgel GF_254_ plates were used for thin-layer chromatography (TLC). Silica gel G_60_ powder was used in column chromatography (CC). Solvents were evaporated under reduced pressure and temperature in a rotavapor. For elemental analysis, a C.H. analyzer was used. FT-IR spectrophotometer (Shimadzu, IR Prestige-21) and Bruker DPX-400 spectrometer were used for IR and NMR scanning, respectively. A CDCl_3_ solution of the sample was used for ^1^H NMR scanning, and the chemical shifts are shown on a delta scale using TMS as the standard.

#### 2.1.1. 1,2:3,4-Di-*O*-isopropylidene-α-d-galactopyranose (G0)

This compound G0 (Fig. S1†) was synthesized from finely powdered dry d-galactose (Gal) and 2,2-dimethoxypropane (32 mL) using *p*-toluenesulfonic acid (*p*-TSA) as catalyst in very good yield as a clear syrup following the reported procedure.^29^

#### 2.1.2. 6-*O*-Acetyl-1,2:3,4-di-*O*-isopropylidene-α-d-galactopyranose (G2)

This compound was prepared from G0 using the literature method.^30^

#### 2.1.3. 1,2:3,4-Di-*O*-isopropylidene-6-*O*-pentanoyl-α-d-galactopyranose (G5)

To a mixture of protected galactopyranose G0 (0.1 g, 0.384 mmol) in pyridine (1 mL) pentanoyl chloride (0.055 g, 0.456 mmol) was slowly added at low temperature. Stirring of this mixture was continued for 10 h at 25 °C. After completion of the reaction as indicated by TLC, concentration of the mixture followed by CC (petroleum ether/ethyl acetate = 15/1) gave the 6-*O*-pentanoate G5 (0.111 g, 84%) as an oil. *R*_f_ = 0.51 (petroleum ether/ethyl acetate = 4/1); FT-IR (CHCl_3_): 1730 (CO), 1381, 1376 cm^−1^ [C(CH_3_)_2_]; ^1^H NMR (400 MHz, CDCl_3_): *δ*_H_ 5.52 (1H, d, *J* = 5.0 Hz, H-1), 4.61 (1H, dd, *J* = 7.6 and 2.5 Hz, H-3), 4.26–4.33 (2H, m, H-2 and H-6a), 4.22 (1H, dd, *J* = 7.6 and 2.3 Hz, H-4), 4.10–4.19 (1H, m, H-5), 4.03 (1H, dd, *J* = 11.2 and 7.4 Hz, H-6b), 2.33 [2H, t, *J* = 7.6 Hz, CH_3_(CH_2_)_2_C*H*_2_CO], 1.56–1.61 [2H, m, CH_3_CH_2_C*H*_2_CH_2_CO], 1.50 [3H, s, C(C*H*_3_)_2_], 1.44 [3H, s, C(C*H*_3_)_2_], 1.32 [3H, s, C(C*H*_3_)_2_], 1.29 [3H, s, C(C*H*_3_)_2_], 1.18–1.24 [2H, s, CH_3_C*H*_2_(CH_2_)_2_CO], 0.87 [3H, t, *J* = 7.3 Hz, C*H*_3_(CH_2_)_3_CO]. Anal. calcd for C_17_H_28_O_7_ (344.40): C, 59.29; H, 8.20. Found: C, 59.36; H, 8.23.

#### 2.1.4. 6-*O*-Hexanoyl-1,2:3,4-di-*O*-isopropylidene-α-d-galactopyranose (G6)

Treatment of protected galactopyranose G0 (0.1 g, 0.384 mmol) with hexanoyl chloride (0.062 g, 0.461 mmol) in anhydrous pyridine (2 mL) for 11 h gave a faster-moving (*R*_f_ = 0.53) compound, which was passed through column chromatography for purification. The product 6-*O*-hexanoyl derivative G6 (0.128 g, 93%) was isolated as a homogeneous syrup. *R*_f_ = 0.53 (petroleum ether/ethyl acetate = 4/1); FT-IR (CHCl_3_): 1734 (CO), 1380, 1375 cm^−1^ [C(CH_3_)_2_]; ^1^H NMR (400 MHz, CDCl_3_): *δ*_H_ 5.50 (1H, d, *J* = 4.8 Hz, H-1), 4.58 (1H, dd, *J* = 7.8 and 2.4 Hz, H-3), 4.27–4.32 (2H, m, H-2 and H-6a), 4.22 (1H, dd, *J* = 7.8 and 2.3 Hz, H-4), 4.16 (1H, dd, *J* = 11.1 and 7.4 Hz, H-6b), 3.95–4.02 (1H, m, H-5), 2.32 [2H, t, *J* = 7.3 Hz, CH_3_(CH_2_)_3_C*H*_2_CO], 1.60–1.64 [2H, m, CH_3_(CH_2_)_2_C*H*_2_CH_2_CO], 1.48 [3H, s, C(C*H*_3_)_2_], 1.42 [3H, s, C(C*H*_3_)_2_], 1.33 [3H, s, C(C*H*_3_)_2_], 1.20–1.27 [7H, br m, CH_3_(C*H*_2_)_2_(CH_2_)_2_CO and C(C*H*_3_)_2_], 0.86 [3H, t, *J* = 7.2 Hz, C*H*_3_(CH_2_)_4_CO]. Anal. calcd for C_18_H_30_O_7_ (358.43): C, 60.32; H, 8.44. Found: C, 60.39; H, 8.42.

#### 2.1.5. 1,2:3,4-Di-*O*-isopropylidene-6-*O*-octanoyl-α-d-galactopyranose (G8)

A mixture of protected galactopyranose G0 (0.1 g, 0.384 mmol) with octanoyl chloride (0.075 g, 0.461 mmol) in pyridine (1 mL) was treated for 12 h at 25 °C. After completion, the mixture was quenched with cold water (0.5 mL), extracted with chloroform, and passed through column chromatography, which furnished the 6-*O*-octanoate G8 (0.116 g, 78%) as an oil. *R*_f_ = 0.56 (petroleum ether/ethyl acetate = 4/1); FT-IR (CHCl_3_): 1738 (CO), 1380, 1372 cm^−1^ [C(CH_3_)_2_]; ^1^H NMR (400 MHz, CDCl_3_): *δ*_H_ 5.52 (1H, d, *J* = 4.6 Hz, H-1), 4.58 (1H, dd, *J* = 7.7 and 2.4 Hz, H-3), 4.27–4.32 (2H, m, H-2 and H-6a), 4.24 (1H, dd, *J* = 7.7 and 2.3 Hz, H-4), 4.14 (1H, dd, *J* = 11.0 and 7.4 Hz, H-6b), 3.93–4.00 (1H, m, H-5), 2.33 [2H, t, *J* = 7.4 Hz, CH_3_(CH_2_)_5_C*H*_2_CO], 1.58–1.63 [2H, m, CH_3_(CH_2_)_4_C*H*_2_CH_2_CO], 1.49 [3H, s, C(C*H*_3_)_2_], 1.44 [3H, s, C(C*H*_3_)_2_], 1.31 [3H, s, C(C*H*_3_)_2_], 1.20–1.29 [11H, br m, CH_3_(C*H*_2_)_4_(CH_2_)_2_CO and C(C*H*_3_)_2_], 0.87 [3H, t, *J* = 7.4 Hz, C*H*_3_(CH_2_)_4_CO]. Anal. calcd for C_20_H_34_O_7_ (386.49): C, 62.16; H, 8.87. Found: C, 62.24; H, 8.90.

#### 2.1.6. 1,2:3,4-Di-*O*-isopropylidene-6-*O*-lauroyl-α-d-galactopyranose (G12)

A mixture of galactopyranose G0 (0.1 g, 0.384 mmol) and lauroyl chloride (0.1 g, 0.457 mmol) was reacted for 14 h. Work-up and CC purification furnished the title compound G12 (0.139 g, 82%) as a solid, mp 129–131 °C. *R*_f_ = 0.58 (petroleum ether/EA = 4/1); FT-IR (CHCl_3_): 1734 (CO), 1382, 1375 cm^−1^ [C(CH_3_)_2_]; ^1^H NMR (400 MHz, CDCl_3_): *δ*_H_ 5.51 (1H, d, *J* = 4.5 Hz, H-1), 4.59 (1H, dd, *J* = 7.6 and 2.4 Hz, H-3), 4.26–4.33 (2H, m, H-2 and H-6a), 4.21 (1H, dd, *J* = 7.6 and 2.3 Hz, H-4), 4.15 (1H, dd, *J* = 11.2 and 7.4 Hz, H-6b), 3.97–4.03 (1H, m, H-5), 2.32 [2H, t, *J* = 7.4 Hz, CH_3_(CH_2_)_9_CH_2_CO], 1.58–1.62 [2H, m, CH_3_(CH_2_)_8_CH_2_CH_2_CO], 1.49 [3H, s, C(C*H*_3_)_2_], 1.43 [3H, s, C(C*H*_3_)_2_], 1.31 [3H,s, C(C*H*_3_)_2_], 1.19–1.32 [19H, br s, CH_3_(C*H*_2_)_8_(CH_2_)_2_CO and C(C*H*_3_)_2_], 0.85 [3H, t, *J* = 7.2 Hz, C*H*_3_(CH_2_)_10_CO]. Anal. calcd for C_24_H_42_O_7_ (442.59): C, 65.13; H, 9.57. Found: C, 65.12; H, 9.62.

### 2.2. DFT optimization details

Gaussian 09W Revision (D.01)17 was employed for geometry optimization and further calculations of synthesized SEs. In the gas phase, a 6-31g+(d, p) basis set combined with B3LYP correlation function and density functional theory (DFT) utilized to identify their physicochemical and spectral features.^31–34^ This basis set was selected due to its proven reliability in similar studies for balancing computational cost with accuracy. The optimized structures are demonstrated in Fig. S2.† Frontier molecular orbitals; ‘Highest Occupied Molecular Orbital’ (HOMO) and ‘Lowest Unoccupied Molecular Orbital’ (LUMO), were determined keeping a similar level of theory. Afterward, the HOMO–LUMO energy gap, chemical softness (*S*), chemical hardness (*η*), electronegativity (*χ*), chemical potential (*μ*), and electrophilicity (*ω*) were determined using the following equations.^35–38^
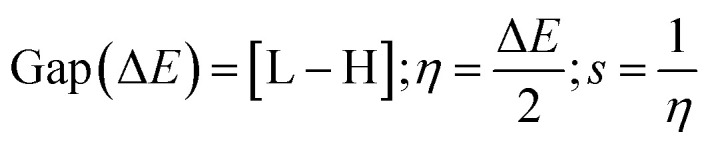

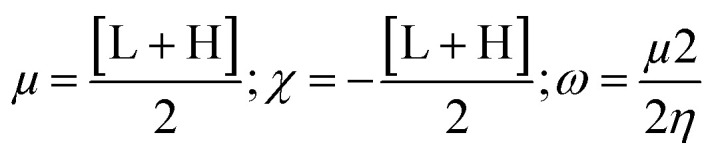
L and H are ‘HOMO’ and ‘LUMO’ energies in eV.

### 2.3. Protein preparation and molecular docking simulation

The crystal structures of COX-1 (1EQG; resolution: 2.61 Å) and COX-2 (5F19; resolution: 2.04 Å) were retrieved from RCSB protein database in PDB format. Both homodimer proteins were imported into BIOVIA Discovery Studio Visualizer software (version 2021) for preparation. The capacity of a substrate to attach to the receptor is frequently unaffected by water molecules. Additionally, crystallized native ligands and unwanted heteroatoms might be present in the receptor's active site. To speed up computations and free up the active site, all the heteroatoms, native ligands, and water molecules were deleted. In COX-2 chain B and in COX-1 chain A were kept, rest were deleted. Swiss PDB Viewer (v.4.10) was utilized for energy minimization of the refined protein structures, where computations were carried out *in vacuo* with the GROMOS 96 force field.

Finally, energy-minimized proteins and previously DFT-optimized galactose esters were subjected to molecular docking utilizing PyRx (version 0.8) virtual screening tools integrated with AutoDock Vina. According to Bauer *et al.*^39^ Autodock Vina showed good screening performance for COX enzymes.^40^ AutoDock Vina in PyRx platform utilized the Lamarckian genetic algorithm (LGA) as a scoring function.^41^ The ligand structures of our studied compounds were first imported into the open babel interface in PyRx. The energy minimization was carried out for the ligands utilizing Universal Force Field (uff). Then they were converted to pbdqt format. Later, site-specific flexible molecular docking was carried out, generating a grid box covered the receptor's active site. The active site of 5F19 was identified from literature study,^28^ and for 1EQG, it was identified from native ligand complexed in its active site. For 5F19, the grid box was set centered at (*X*, *Y*, *Z* = 21.10 Å, 47.90 Å, 19.10 Å) and had a size of 18.53 Å, 31.82 Å, and 23.39 Å in the *x*, *y*, and *z*-axis directions, respectively. For 1EQG grid box, the parameters were: center (26.82 Å, 33.48 Å, 200.84 Å), size (14.54 Å × 9.50 Å × 26.24 Å). The output files generated after the completion of docking were utilized to prepare ligand–protein complexes using PyMol (version 2.5.0). Later, these complexes were used for active site visualization, non-bonding interaction (NBI) analysis, and hydrogen bond surface mapping utilizing BIOVIA Discovery Studio Visualizer software (version 2021).

### 2.4. Docking protocol validation

To check the precision and reproducibility of the docking, we implemented a systematic validation approach encompassing redocking and superimposition techniques. To validate the docking of COX-2 (PDB ID: 5F19), we redocked one of the reference drugs, aspirin, into the designated active site using the same docking parameters. Subsequently, we assessed the RMSD value of the re-docked conformer in comparison to the lowest energy conformer of previously docked aspirin, followed by their superimposition using PyMol. For docking validation of COX-1 (PDB ID: 1EQG), its co-crystal ligand, ibuprofen, was separated and subsequent docking was carried out using the same docking parameters stated for 1EQG. The final lowest energy conformers of both the docked and co-crystal ibuprofen were aligned in PyMOL to calculate the executive root-mean-square deviation (RMSD) value. Typically, an RMSD value of ≤2 Å or 0.2 nm indicates a reliable docking method.^42^

### 2.5. Field-based 3D-QSAR model for p*K*_i_ prediction

A QSAR model quantitatively assesses the relationship between the molecular structures of a series of compounds and their corresponding biological activities. The 3D-QSAR method operates under the assumption that compounds exhibiting structural or physicochemical similarities are likely to demonstrate similar biological activities. A collection of 22 structurally diverse COX-1 inhibitors, with inhibition constant (*K*_i_) values spanning from 7 to 9300 nM, was obtained from the BindingDB database^43^ (Table S3†). Additionally, 25 COX-2 inhibitors, exhibiting a broad spectrum of biological activity with *K*_i_ values ranging between 3 and 14 790 nM, were also compiled (Table S5†). The structures of the molecules were processed using the LigPrep module of Maestro Version 12.5.139 with the following parameters: (i) the OPLS3e force field was applied; (ii) the ionization state was neutral; (iii) chiralities were determined from the 3D structure; and (iv) one low-energy conformer per ligand per ligand was generated. The alignment of molecules is the most essential input for creating a highly predictive field-based 3D-QSAR model. Hence all the LigPrep output molecules were subjected to flexible ligand alignment. Alignment procedure was carried out separately for COX-1 and COX-2 inhibitors, aiming to develop two field based 3D-QSAR models. During flexible ligand alignment, reference structure was chosen automatically. A thorough sampling method was adopted with a maximum number of conformers of 1000 and a nonbonded close contact distance of 0.5 Å.

Two field-based 3D-QSAR models were developed using aligned molecular structures. Both datasets demonstrate diversity in their pharmacological and structural properties. The *K*_i_ values (nM) were converted to p*K*_i_ using the standard conversion formula: p*K*_i_ = −log(10^−9^ × *K*_i_). Each dataset was randomly partitioned into a training set (70%) and a test set (30%), with a partial least squares (PLS) factor of 3 applied. The random selection performed by the software was subsequently verified through visual inspection to ensure that the training and test sets maintained diversity. In this study, interaction energy calculations for the 3D QSAR model were conducted utilizing five different fields such as steric, electrostatic, hydrophobic, hydrogen bond donor (HBD), and hydrogen bond acceptor (HBA). These calculations were performed using Gaussian equations to evaluate the respective fields.^44^ The optimal model was chosen based on its statistical robustness. Validation was conducted using a test set comprising 30% compounds of both datasets. Key parameters for evaluating the test set included RMSE, *Q*^2^, and Pearson's *r* (Tables S7 and S9†), which collectively reflect the model's predictive accuracy. To prevent overfitting, the number of PLS factors was carefully limited to 3. Additionally, scatter plots were generated to illustrate the correlation between the observed and predicted activities of the dataset molecules (Fig. S6†).

### 2.6. ADMET, PASS, and drug-likeness prediction

For all studied compounds along with standard ligands, some pharmacokinetic properties (ADMET, PASS, and drug-likeness) were assessed to predict their drug-ability. ADMET features play a key role in drug development, with half of the drug candidates failing since they lack the pharmacokinetic features.^45^ The AdmetSAR web server was used to evaluate each of the compounds' ADMET profile.^46^ Selected pharmacokinetic properties, *i.e.*, human intestinal absorption, inhibition of cytochrome P450, Blood–Brain Barrier (BBB) penetration, *etc.*, were considered for the evaluation.

Using Lipinski's rule of five, substances similar to drugs and those not were distinguished. The SwissADME server was used to estimate crucial pharmacokinetic parameters such as molecular weight, hydrogen bond donor, hydrogen bond acceptor, TPSA (topological polar surface area), and consensus log *P*o/w. Lipinski's Rule was also predicted using the same web server.^47^ Some probable pharmacological properties of the SEs were studied using the PASS online tool to reveal their therapeutic possibilities as well as their adverse effects.^48^

### 2.7. Molecular dynamics simulation

We used molecular dynamics (MD) simulation, which uses Newton's law of motion to mimic the behavior of the actual biological environment, including macromolecules or more complex systems.^49^ Using the Desmond module in Schrödinger Suite (2017-1) software, a 100 nanosecond molecular dynamics simulation (MDS) was carried out to assess the stability and compactness of the docked complex. G6 and G8 complexed with 5F19 were chosen for the simulation. Asp-protein and Rxb-protein complexes were also subjected to MDS as references for comparison. The complexes were derived from molecular docking investigations. The System Builder tool was used to prepare the system. In the simulation, the OPLS_2005 force field was employed.^50^ Under orthorhombic periodic boundary conditions for the 10 Å buffer zone, the molecular system was solvated using crystallographic water (TIP3P).^51^ After removing the overlapping water molecules, the system was neutralized by introducing 0.15 M NaCl to add 3 Cl-ions as counterions. The neutralized systems were subjected to energy minimization using the steepest descent algorithm at 1000 steps. An ensemble (NPT) of Nose–Hoover thermostats and barostats was used for system equilibration, maintaining the system's constant temperature (300 K) and pressure (1 bar).^52^ Finally, 100 ns production MDS was run for each system and Trajectories within 25 ps intervals were recorded for analysis throughout the 100 ns simulation time, which yields about 4000 frames. These trajectories were further utilized to analyze root mean square deviation (RMSD), root mean square fluctuation (RMSF), solvent accessible surface area (SASA), and total contacts.

## 3 Results and discussion

### 3.1. Synthesis of 6-*O*-acylgalactopyranoses

**Scheme 1:**
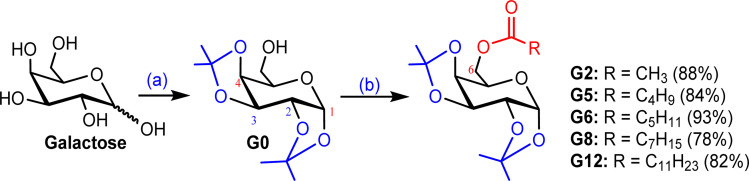
(a) 2,2-Dimethoxypropane, *p*-TSA, 60–70 °C, 5 h, 89%; (b) dry Py, CH_3_COCl/C_4_H_9_COCl/C_5_H_11_COCl/C_7_H_15_COCl/C_11_H_23_COCl, 0–25 °C, 10–12 h.

Our main aim was to introduce strain and acyl/ester group in the galactose molecule so that it can easily migrate an acyl group to the enzyme to minimize its molecular strain. As galactopyranose has 5 hydroxyl groups, its bisacetonation gave 1,2:3,4-bis acetonide and introduced strain in the molecule. Subsequently, acylation (esterification) gave C-6 ester only. Thus, we exploited C-6 esters of d-galactose. As shown in Fig. S1,† the attachment of the ester (non-sugar) group at the C-6 position of d-galactose enhanced its biological profile. Initially, protected galactopyranose G0 was prepared from galactose in 89% yield using a literature procedure (Scheme 1). Having G0 in hand, its free hydroxyl at the C-6 position was esterified with five different fatty acyl halides (2C to 12C) employing the direct method. All these 6-*O*-acyl products were characterized by spectral methods.

The conformational behaviors of biologically active compounds are an important component for interactions with proteins' receptors.^53^ Thus, the conformations of all the synthesized esters were studied from their coupling constants (^1^H NMR) and presented in Table 1. Galactose with regular ^4^*C*_1_ chair conformation showed the usual coupling constant (*J*) between *trans* (H-2, H-3) and *cis* (H-3, H-4) coupled protons as 5.96 (*J*_2,3_) and 5.08 (*J*_3,4_) Hz, respectively.^29^ However, incorporation of two fused five-membered isopropylidene rings in galactose, as in G0, shifted to 2.40 (*J*_2,3_) and 7.90 (*J*_3,4_) Hz, respectively (Table 1), indicating that galactose changed its conformation from chair to twist-boat.^30^ Further addition of acyl group in G0 at the C-6 position, as in G2–G12, changes coupling constants at *J*_2,3_, *J*_3,4_ and *J*_4,5_ positions (Table 1) from regular chair conformation, resulting in these molecules (G2–G12) being more twisted or strained.

**Table 1:** Unusual coupling constant between protons

Galactose compounds	Coupling constant (*J*), Hz			
	*cis*, *J*_1,2_	*trans*, *J*_2,3_	*cis*, *J*_3,4_	*cis*, *J*_4,5_
G0^a^	5.0	2.40	7.90	1.30
G2	—	2.40	7.90	—
G5	5.00	2.50	7.60	2.30
G6	4.80	2.40	7.80	2.30
G8	4.60	2.40	7.70	2.30
G12	4.50	2.40	7.60	2.30
*J* (calcd) chair^b^	4.66	5.96	5.08	1.19
*J* (calcd) twist-boat^b^	4.66	2.93	7.79	1.98

a Reported data.^30^

b Reported data.^29^

### 3.2. Frontier molecular orbitals (FMOs) and chemical reactivity descriptor analysis

**Fig. 1:**
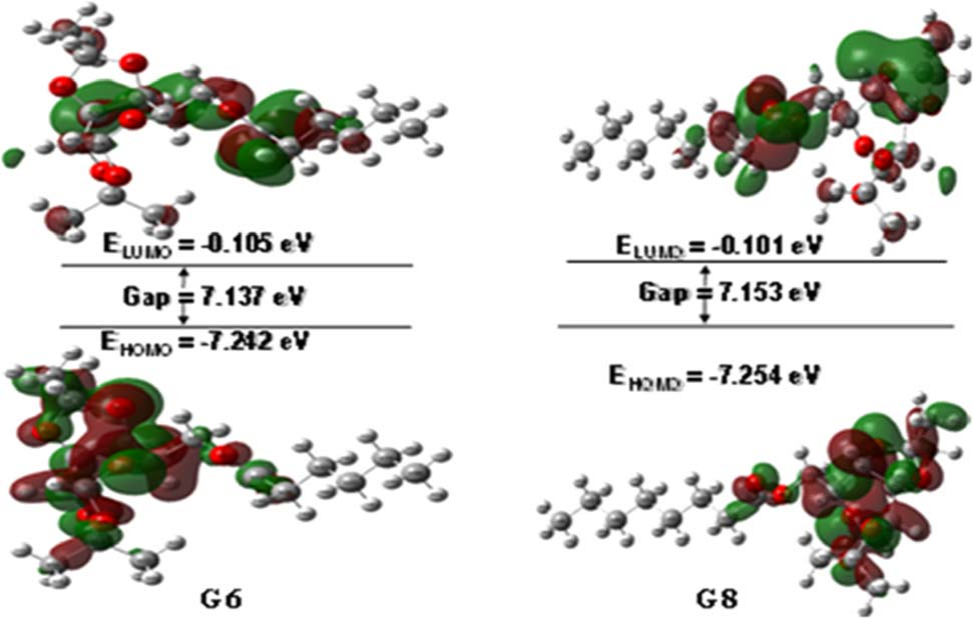
HOMO–LUMO orbitals with their energy gap.

The interaction of a molecule with other species is prevailed by the FMOs; HOMO and LUMO.^54^ The FMO energy gap helps in describing the molecule's reactivity and stability. A soft molecule has a small energy gap in its FMO, which makes it more polarizable and often indicates strong chemical reactivity and low kinetic stability. On the other side, a more stable molecule emerges when the energy gap is larger.^55^ The energy gap of the compounds was determined to be between 6.58 eV and 7.17 eV, as depicted in Fig. S3 (Table S1),† suggesting that the studied esters are stable. Fig. 1 illustrates the 3D plot of the investigated molecule's HOMO and LUMO frontier orbitals with their corresponding energy gaps (Fig. S4†). The negative phase is green, while the positive phase is red. The following compounds are listed in order of decreasing stability: G5, G8, G12, G6, G0, G2, and Gal, and their reactivity is in the opposite sequence. All the synthesized esters are presumed to exhibit greater kinetic stability than standard aspirin and ibuprofen due to their higher Frontier Molecular Orbital (FMO) energy gaps.

### 3.3. Molecular electrostatic potential (MEP) map analysis

**Fig. 2:**
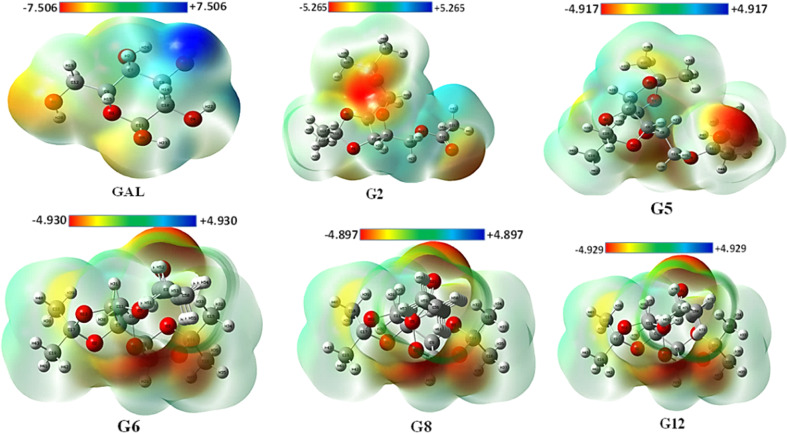
MEP with their positive and negative potentiality.

The MEP may be used to interpret hydrogen bonding and the biological recognition process.^56^ For all optimized structures, the reactive sites for electrophilic and nucleophilic attacks were predicted using the MEP (Fig. 2). The positive region in blue is an appropriate site for nucleophilic attack, while the maximum negative area in red is a favorable site for electrophilic strike, and the zero potential area is depicted in green in Fig. 2 (also in Fig. S5†). While the blue area lacks electrons, the red region is electron-rich. The maximum positive potentiality is found for hydrogen atoms (H atoms depicted in white color), whereas the highest negative potentiality is found for oxygen atoms (red-colored atoms). In this current investigation, Gal exhibited the highest positive potentiality (+7.5 a.u.) and also showed the maximum negative potentiality of −7.5 a.u. G8 exhibited the lowest positive (+4.897 a.u.) and negative (−4.897 a.u.) potentiality among the compounds. Due to Gal's increased electrostatic potentiality, it shows the highest number of hydrogen bonding interactions with the target macromolecule (Table S2†). Asp and Ibp also display notable electrostatic potentials of ±7.352 a.u. and ±6.140 a.u., respectively (Fig. S4†), which contribute to their ability to form additional hydrogen bonds with the receptor, as detailed in Table S2.†

### 3.4. Docking validation results

In molecular docking, it's essential to accurately determine where the ligand fits into the structure of the target protein. A pivotal metric for evaluating this process's precision is the root mean square deviation (RMSD), which quantifies the mean distance between atoms within superimposed complexes, reflecting the resemblance between the docked and re-docked structures. A smaller RMSD value indicates a higher degree of similarity between the docked pose and the reference structure, suggesting the reliability and reproducibility of the docking methodology.

**Fig. 3:**
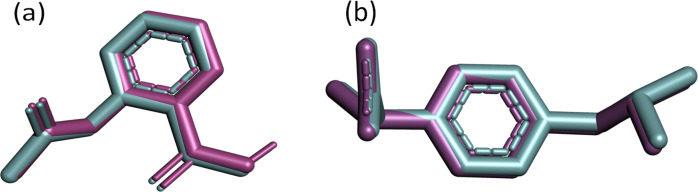
(a) Superimposition of docked (magenta) and re-docked (cyan) aspirin in the COX-2 active site with an RMSD of 1.61 Å; (b) aligned cocrystal structure (magenta) and re-docked conformer (cyan) of ibuprofen with an RMSD of 1.399 Å in the active site of COX-1.

In the superimposition analysis of aspirin molecules docked and re-docked poses within the COX-2 active site, an initial assessment involved comparing 14 atoms from each conformer using pairwise scoring. Throughout the refinement process, multiple alignment cycles were executed without excluding any atoms, ultimately leading to a final alignment involving all 14 atoms present in each structure. The resultant executive RMSD value was 1.61 Å, suggesting an exceedingly similar alignment between the docked and re-docked structures, with negligible deviations observed (Fig. 3(a)).

The superimposed pose of cocrystal and re-docked conformations of ibuprofen residing in the COX-1 active site are depicted in Fig. 3(b). Following the alignment of 15 atoms of each ibuprofen structure, it gives an RMSD value of 1.399 Å.

The observed root mean square deviation (RMSD) for both COX-1 and COX-2 was below 2 Å, falling within an acceptable range. Overall, these results highlight the accuracy of the docking methods, with the docked, cocrystal, and re-docked conformers closely matching their respective complexes. We can infer that using the same docking parameters will likely yield accurate positions for the studied galactose derivatives.

### 3.5. Molecular docking: binding affinity and non-bonding interactions studies

The molecular docking scores of our investigated esters are shown in Table 2 alongside two common NSAIDs: aspirin (Asp) and ibuprofen (Ibp). For better comparision, we carried out molecular docking of selective COX-2 inhibitor, Rofecoxib (Rxb) as well. The larger negative value depicts the strong interaction between ligands and receptors. The synthesized esters (G2, G5, G6, and G8) in our current investigation showed a greater binding affinity for the receptor protein, 5F19. They exhibited better docking scores than standard drugs. Among the compounds, G8 showed the highest binding affinity, with a binding energy equal to −10.4 kcal mol^−1^. The binding interactions of other ester derivatives; G2, G5, G6, and G12, were −9.7, −9.7, −10.1, and −2.3 kcal mol^−1^, respectively. The drastic decline in the binding affinity of G12 may prevail that the dodecanoyl chain attached to G12 is too large to occupy the space available in the active site cavity of the receptor. Thus, it can be said that the octanoyl group attached to G8 is the optimal ester chain that can exert high inhibitory activity by blocking the active site for its substrate. We modelled another hypothetical SE compound, G10, having a decanoyl group at the C-6 position. We docked the compound with the receptor following the same docking protocol to test our assumption. It results in a binding affinity of −8.8 kcal mol^−1^, suggesting a trend in the increase in docking score with the increase in acyl group chain length constrained until G8, comprising the octanoyl group.

**Table 2:** Binding affinity and predicted p*K*_i_ value of galactose derivatives and the standard drugs with the receptors COX-2 (5F19) and COX-1 (1EQG)

Ligands	COX-2 (5F19)		COX-1 (1EQG)	
	Binding affinity (kcal mol^−1^)	Predicted p*K*_i_ value	Binding affinity (kcal mol^−1^)	Predicted p*K*_i_ value
Gal	−6.7	6.388	—	5.579
G0	−8.6	6.302	—	5.247
G2	−9.7	6.465	−6.5	5.531
G5	−9.7	6.407	−2.0	4.795
G6	−10.1	6.504	0.3	5.224
G8	−10.4	6.973	5.8	5.079
G10	−8.8	5.764	—	5.713
G12	−2.3	5.995	—	4.860
Asp	−7.4	5.753	−7.0	5.376
Ibp	−9.0	6.422	−8.1	5.604
Rxb	−9.4	6.340	−4.4	6.564

The stability of a drug within the target site is also determined by non-bonding interactions, which also increase the ability and alter the binding affinity. Some types of non-covalent interactions are carbon–hydrogen, conventional hydrogen, and hydrophobic bonds. All of these interactions were observed in our present study (Table S2†). Parent galactose (Gal) showed a maximum number (three) of H bonds with the AAs; LEU531, and GLY533. It is because of the presence of the polar OH group. However, it lacks the presence of the hydrophobic interaction, resulting in lower binding affinity (−6.7 kcal mol^−1^). Protected galactose G0 showed different magnitudes of hydrophobic interaction within the active site and also an increase in docking score (−8.6 kcal mol^−1^). Again, when the acyl group was introduced to the protected galactose G2, its binding affinity further increased. Also, the number of hydrophobic interactions between target and ligand increased.

**Fig. 4:**
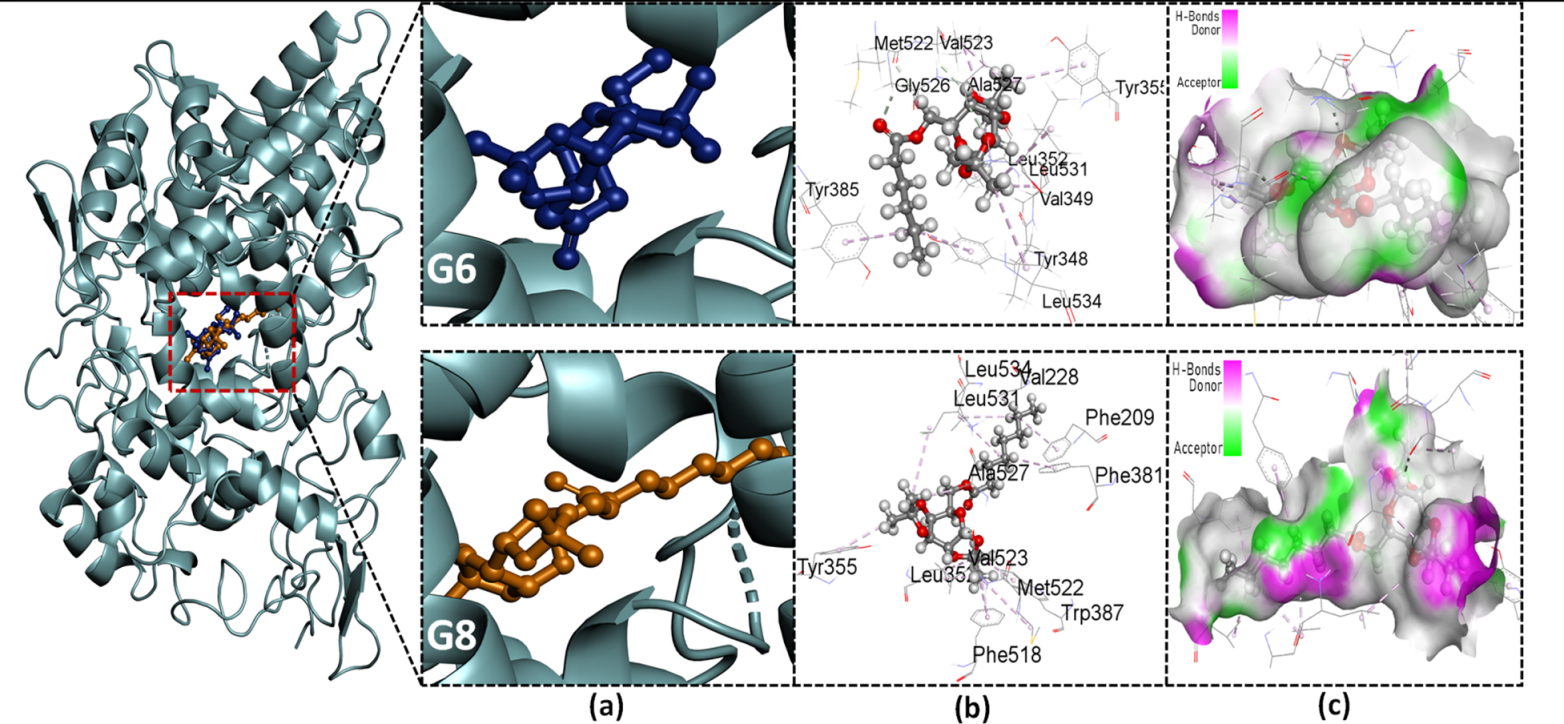
Docked conformer of the synthesized esters (G5, G6, G8) interacting in the same binding pocket of receptor protein: (a) zoomed pose; (b) 3D depiction of non-bonding interaction with amino acid residues; (c) hydrogen bond surface area.

There is a secondary-binding pocket and a hydrophobic pocket in the active site of COX-2. Compounds that can particularly bind in the secondary pocket and provide enough steric bulk to block COX-2's hydrophobic channel are essential for extremely effective and selective COX-2 inhibition.^28^ The key amino acid (AA) residues in the secondary pocket are Val523, Ala516, Tyr355, Ser353, and Ala527. The hydrophobic pocket has the AA residues; Tyr385, Phe518, Phe381, Leu352, Leu384, Trp387, and Met522.^28^ Again, Ser530 and Tyr 385 are two important AAs for the acylation of the enzyme, thus its inhibition by aspirin.^57^ From the NBI analysis (Fig. 4, S6 and Table S2†) of the docked complexes, it was observed that all of our studied SEs interact with these AAs with different bond distances. These interactions suggest that the studied SEs can fit into both a secondary pocket and a hydrophobic pocket in the active site, which may block the substrate binding to the enzyme, thus inhibiting its activity.

To evaluate the specificity of our synthesized esters for COX-2 over COX-1, they were further subjected to molecular docking with the COX-1 isoenzyme. It has already been discussed that the substrate-binding pocket of COX-1 is smaller than that of COX-2. Table 2 depicts the ligand's binding affinity for COX-1. Among the studied SEs, G2 showed a maximum binding affinity of −6.5 kcal mol^−1^ for COX-1. Afterward, the affinity of compounds with a higher acyl chain drastically falls. From G6, it starts to give a positive value of binding affinity, implying the compounds with bulk acyl chains cannot bind to the active site spontaneously. This finding may facilitate the design of selective COX-2 inhibitors.

### 3.6. Field-based 3D-QSAR statistical results and p*K*_i_ prediction

The field-based 3D-QSAR model was developed by randomly partitioning the two datasets into 70% for the training set and 30% for the test set with applying PLS factor 3. As demonstrated in Tables S7 and S9,† the best model (PLS factor = 3) exhibited strong predictive capability for both the COX-1 and COX-2 inhibition datasets. A QSAR model is considered acceptable when it demonstrates an *R*^2^ value exceeding 0.6, an *R*_CV_^2^ value greater than 0.5, a high *F*-statistic, and a low *p*-value.^58^ Among the three models generated for both datasets, the third model, with a PLS factor of 3, demonstrated statistically significant performance metrics. Specifically, the models achieved higher *R*^2^ values of 0.9114 for COX-1 and 0.9400 for COX-2, indicating robust correlation between predicted and observed values. Additionally, the model exhibited favorable cross-validated *R*_CV_^2^ values of 0.4975 for COX-1 and 0.8404 for COX-2, suggesting reliable predictive capability. The *F*-values were also substantial, with 41.1 for COX-1 and 73.1 for COX-2, underscoring the model's statistical significance. Furthermore, the model demonstrated lower RMSE values of 0.71 for COX-1 and 0.45 for COX-2, reflecting its precision in predicting inhibitory activities (refer to Tables S7 and S9†). The fractions of five Gaussian factors contributed in the models are enlisted in Tables S8 and S10.† Scatter plots correlating the experimental and predicted activities for the datasets are presented in Fig. S6.† The plots indicate a satisfactory level of scattering, suggesting a reasonable agreement between the experimental and predicted values.

Finally, utilizing the two QSAR models, the p*K*_i_ values of the synthesized esters were calculated (see Table 2). The results indicate that these esters exhibit superior inhibition constants for COX-2 compared to COX-1. This finding suggests a preferential selectivity of the esters for COX-2, a trend that is corroborated by the molecular docking studies.

### 3.7. Pharmacokinetics: ADMET analysis

ADMET screening of drugs has garnered significant interest for its ability to mitigate the extensive costs and time associated with *in vivo* experiments. This approach offers a streamlined means of drug development by examining absorption, distribution, metabolism, excretion, and toxicity properties. According to the ADMET calculation as depicted in Table 3, all the SEs have higher human intestinal absorption (HIA). It is seen that protected acylated galactose derivatives have a higher HIA rate than parent galactose. Greater HIA suggests that certain drugs, following oral administration, have better intestinal absorption power. Most of the SEs (G5, G6, G8, and G12) with the increase in acyl chain length showed CACO-2 permeability, while parent (Gal) and protected galactose (G0) were seen to be impermeable. All the studied compounds, as well as the reference compounds, showed positive outcomes in terms of crossing the blood–brain barrier. All SEs showed non-inhibitory activity for human ether-a-go-go related gene, making them relatively safer in terms of cardiotoxicity. hERG inhibition of potassium channels mainly leads to QT syndrome, leading to ventricular arrhythmia.^59^ For this reason, many drugs were restricted in use. Ibp is slightly carcinogenic (probability to be active = 0.53980), whereas the studied SEs were non-carcinogenic. Asp showed category II acute oral toxicity, whereas the rest of the compounds exhibited category III oral toxicity. Therefore, it is evident from our analysis that our synthesized compounds are relatively safer for oral administration. Our study further showed acylated galactoses are P-glycoprotein inhibitors, which may influence their absorption and excretion. The LD_50_ values of the SEs were satisfactory compared to the reference drugs. Galactose derivatives were non-biodegradable, whereas parent galactose and the reference compounds seemed to be readily biodegradable.

**Table 3:** ADMET properties of SEs along with standard drugs^a^

Name	Absorption		Distribution		Metabolism	Toxicity				
Compound	HIA	C2P	P-GI	BBB	CYP4502C9	hERG	Carcinogen	AOT	RAT (LD_50_, mol kg^−1^)	Biodegradation
Gal	−0.7683	−0.8659	NI (0.949)	+0.6433	−0.9656	NI (0.9522)	NC (0.9604)	IV	0.8753	0.9261
G0	+0.9162	−0.5454	NI (0.7816)	+0.9390	−0.9247	NI (0.9632)	NC (0.8400)	III	2.0965	−0.8104
G2	+0.9700	−0.5697	I (0.5087)	+0.9403	−0.8723	NI (0.9579)	NC (0.8449)	III	2.1521	−0.8271
G5	+0.9914	+0.5080	I (0.6505)	+0.9466	−0.7088	NI (0.8715)	NC (0.8680)	III	2.1236	−0.8667
G6	+0.9882	+0.5088	I (0.5087)	+0.9403	−0.8723	NI (0.9579)	NC (0.8449)	III	2.0862	−0.8701
G8	+0.9882	+0.5088	I (0.6500)	+0.9578	−0.6787	NI (0.8052)	NC (0.8635)	III	2.0862	−0.8271
G12	+0.9882	+0.5088	I (0.6500)	+0.9578	−0.6787	NI (0.8052)	NC (0.8500)	III	2.0862	−0.6250
Asp	+0.9645	−0.6607	NI (0.9118)	+0.9376	−0.9071	NI (0.9799)	NC (0.8356)	II	2.6386	0.9067
Ibp	+0.9947	+0.8866	NI (0.9323)	+0.9619	−0.9305	NI (0.9734)	C (0.53980)	III	2.3092	0.5142
Rxb	+0.9938	−0.5696	NI (0.9220)	+0.6296	+0.6668	NI (0.8932)	NC (0.5754)	III	2.4527	−0.7716

a HIA = human intestinal absorption, C2P = CACO-2 permeability, P-GI = P-glyco-protein inhibitor, BBB = blood–brain barrier, hERG = human ether-a-go-go related gene, C = carcinogen, AOT = acute oral toxicity, RAT = rat acute toxicity, NI = non-inhibitor, NC = non-carcinogen.

### 3.8. Pharmacokinetics: drug-likeness and PASS prediction

The “rule-of-five”, Lipinski, and coworkers at Pfizer (Groton, NJ, USA) created after examining 2245 medicines from the WDI believed to have entered Phase II trials. The rule-of-five results in an alert (indicating potential absorption issues) for compounds when any two of the following requirements are met: (1) molecular mass >500; (2) more than 10 hydrogen-bond acceptors; (3) more than 5 hydrogen-bond donors; (4) calculated log *P* >5.0 (when using *C* log *P*) or >4.15 when using *M* log *P*; (5) number of rotatable bonds (NBR) is greater than three.^60^ The Lipinski rule violations, HBD, HBA, NBR, MW, log *P*o/w, log *S*, and derivatives of galactose, along with standard drugs, are all demonstrated in Table 4. All the analyzed galactose derivatives were found to have no violation of the Lipinski rule. According to this study, all these drugs have topological polar surface areas (TPSA) that are higher than 65 Å^2^, and those have higher values than Asp and Ibp. The partition factor (log *P*o/w) of *n*-octanol with water is an important physicochemical factor for the synthesis of new drugs. It is described as the proportion between the concentration of the non-ionized substance in the organic and aqueous components at equilibrium. All the compounds under investigation have optimal lipophilicity.

**Table 4:** Comparative drug likeness parameters of SEs to standard drugs^a^

Name	HBD	HBA	NBR	TPSA (Å^2^)	Log *P*o/w	Log *S* (mg ml^−1^)	MW (g mol^−1^)	Lipinski rule	
								Follow	Violation
Gal	5	6	1	110.38	−2.26	1.15	180.16	5	0
G0	1	6	1	66.38	0.63	−1.38	260.28	5	0
G2	0	7	3	72.45	1.15	−1.87	302.32	5	0
G5	0	7	6	72.45	2.25	−2.80	344.40	5	0
G6	0	7	7	72.45	2.59	−3.16	358.43	5	0
G8	0	7	9	72.45	3.31	−3.88	386.48	5	0
G12	0	7	13	72.45	4.76	−5.33	442.59	5	0
Asp	1	4	3	63.60	1.28	−1.85	180.16	5	0
Ibp	1	2	4	37.30	3.00	−3.36	206.28	5	0
Rxb	0	4	3	68.82	2.79	−3.42	314.36	5	0

a HBD = number of H-bond donors, HBA = number H-bond acceptors, NBR = number of rotatable bonds, TPSA = topological polar surface area, MW = molecular weight.

Some selected biological activity parameters of the ester derivatives, along with the reference drugs, are tabulated in Table 5. The synthesized esters exhibited greater predicted anti-inflammatory activity compared to common NSAIDs such as aspirin (Asp), ibuprofen (Ibp), and rofecoxib (Rxb). The SEs also exhibited lower gastrointestinal toxicity than Asp, Ibp, and Gal. Our studied esters also seemed to be less toxic in hepatitis, nephrotoxicity, hematotoxicity, and neurotoxicity parameters. Therefore, it may be said that acylated sugar has a lower toxicity profile than parent sugar and standard drugs.

**Table 5:** Predicted biological activity of all sugar esters

Name	Anti-inflammatory	Gastrointestinal toxicity	Hepatitis	Nephrotoxic	Hematotoxic	Neurotoxic
Gal	0.702	0.863	0.722	0.756	0.855	0.853
G0	0.983	0.596	0.577	0.722	—	0.567
G2	0.981	0.549	0.539	0.746	0.407	0.385
G5	0.966	0.572	0.481	0.702	0.427	0.445
G6	0.966	0.572	0.481	0.702	0.427	0.445
G8	0.966	0.572	0.481	0.702	0.427	0.445
G12	0.966	0.572	0.481	0.702	0.427	0.445
Asp	0.733	0.713	0.406	0.640	0.534	0.233
Ibp	0.901	0.744	0.817	0.745	0.734	0.693
Rxb	0.828	—	—	—	—	—

### 3.9. Molecular dynamics simulation and analysis

**Fig. 5:**
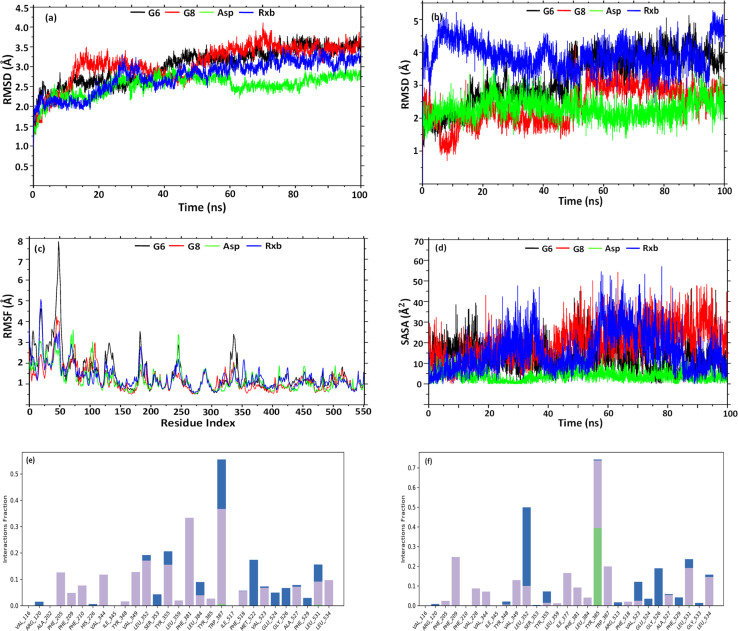
Graph representation of RMSD of (a) backbone; (b) ligand–fit protein; (c) RMSF of backbone; (d) solvent accessible surface area of ligands, and histogram of protein AA contacts with G6 (e) and G8 (f) throughout the simulation time.

Having better binding affinities for SEs, it was aimed at checking the stability, and compactness of the protein–ligand complexes, including molecular insights involved in the binding of G6, G8, Asp, and Rxb in the active pocket of 5F19. Hence, a 100 ns molecular dynamic simulation of the complexes was performed. The plot in Fig. 5(a) shows the RMSD evolution of the protein backbone in the complexes. Following the alignment of each protein frame on the reference frame backbone, atom selection is used to compute the RMSD. Throughout the simulation, tracking the protein's RMSD can provide insight into its structural conformation. From the trajectory analysis, the RMSD of the protein backbone was found in the range of 1.25–3.7 Å (G6 and G8), 1.25–2.75 Å (Asp), and 1.25–3.48 Å (Rxb), respectively. The G6 complex was stabilized after 40 ns and maintained its consistency in RMSD within 3–3.5 Å till the end of the simulation time. G8 was stabilized after 50 ns and maintained its stability with a similar trend in its RMSD value. Asp showed a lower RMSD and maintained its consistency from the beginning of the trajectories. A similar trend in RMSD was observed for the complex of selective COX-2 inhibitor Rxb. Ligand RMSD depicts its stability with protein in the binding pocket of protein. Fig. 5(b) indicates the RMSD of the ligand as the protein–ligand complex is first aligned on the protein backbone of reference. The graph indicates that after 50 ns, the G6 and G8 ligands were well-stable when fitting into the active site cavity. After 50 ns, G6 showed the RMSD ranging between 3.5 and 4.5 Å, while G8 showed slightly lower (2.5–3.5 Å). The standard ligand, Asp, was stable from the beginning of the trajectories, with an RMSD value ranging between 1.5 and 3 Å. Another standard ligand, Rxb, showed overall higher and inconsistent ligand RMSD than the synthesized compounds (Fig. 5(b)).

The root mean square fluctuation (RMSF) helps define local alterations of the amino acid residues along the protein chain, thus determining the flexibility of residues in ligand binding. In Fig. 5(c), peaks indicate the AAs of the receptor that fluctuate the most throughout the trajectories. The complex of G6 shows slightly higher fluctuation in RMSF values than the G8, Asp, and Rxb complexes. The trend in RMSF values was almost identical in the three complexes. Ligand's solvent accessible surface area (SASA) was analyzed to predict the change in ligand surface area while complexed with the receptor protein. Higher SASA indicates the expansion of surface, whereas lower values depict their truncated state. As shown in Fig. 5(d), G8 tends to gain an elevated surface after 50 ns of the simulation and was observed to have a consistent deviation throughout the rest of the simulation. G6 also tends to be consistent in the deviation of its surface area throughout the trajectories. Asp showed the lowest SASA values during the simulation. Higher SASA can be correlated with the strained nature and bulk acyl group of the esters. Rxb exhibited considerable variability in its SASA values across the trajectories. Fig. 5(e) and (f) illustrate the histogram of the interaction fraction of the amino acid residues during the simulation with G6 and G8, respectively, which agrees well with the results of docking.

## 4 Conclusions

The most commonly prescribed non-steroidal anti-inflammatory drugs (NSAIDs) that act on COX have several side effects. Due to some structural resemblance with COX inhibitor aspirin (six-membered ring and acyl group), several synthesized strained SEs are evaluated for their COX inhibition. Encouragingly, two SEs (G6 and G8) have a higher binding affinity for COX-2 but a lower one for COX-1 because the active site pocket volume of COX-2 is larger compared to COX-1. The fact is also supported by the superior inhibition constants (3D-QSAR statistical results with p*K*_i_ prediction) for COX-2 compared to COX-1 and suggests a preferential selectivity of the esters for COX-2. Additional support of such significant binding affinity and selectivity, MD simulation (100 ns), ADMET, drug-likeness, and PASS prediction were also carried out and discussed. Hopefully, the study will encourage future applications of SEs as NSAIDs.

## Data availability

Samples and spectra of the compounds are available from the authors. All the analyzed data are presented in the manuscript. Computational programme and software are also mentioned in the manuscript. Also, the following ESI can be downloaded at as ESI Fig. S1–S6 and Tables S1–S10.†

## Conflicts of interest

The authors declare no conflict of interest. The funders had no role in the design of the study; in the collection, analyses, or interpretation of data; in the writing of the manuscript; or in the decision to publish the results.

## Supplementary Material

RA-014-D4RA03520H-s001
