# Supplementary material for: Synthesis and elucidation of strained galactopyronose esters as selective cyclooxygenase-2 inhibitor: a comprehensive computational approach
Source: RSC Adv. 2024 Sep 24;14(41):30469–81. doi: 10.1039/d4ra03520h (PMC11421415; doi:10.1039/d4ra03520h)
Supplement: RA-014-D4RA03520H-s001 [file RA-014-D4RA03520H-s001.pdf]

Supplementary Information (SI) for RSC Advances.
This journal is © The Royal Society of Chemistry 2024

Supplementary Information

Synthesis and elucidation of strained galactopyronose esters as selective cyclooxygenase-2 inhibitor: A comprehensive computational approach

Mohammed Sakib Musa[a], Md. Sopon Miah[b], Yeasmin Akter Munni[c], Md. Abdul Majed Patwary[d], Mohsin Kazi[e]*, Mohammed Mahbubul Matin[b]*

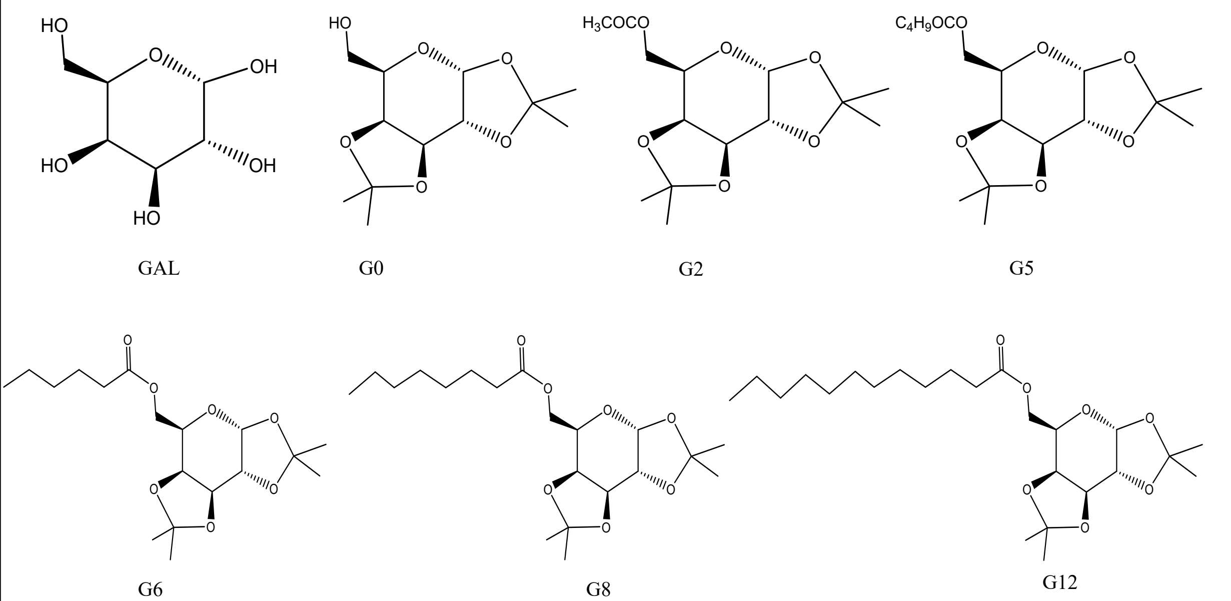

Figure S1. Structure of galactose esters.

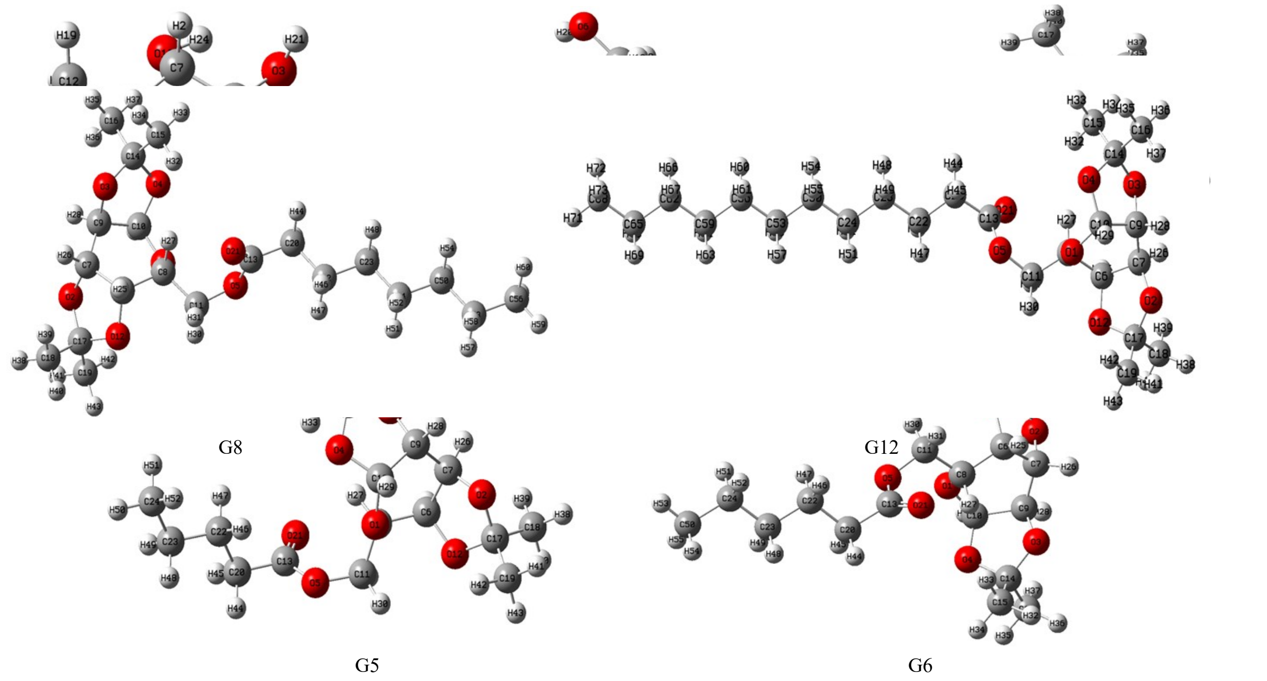

Figure S2. Optimized [DFT method under B2LYP/6-31g+(d,p)] structures of galactose esters.

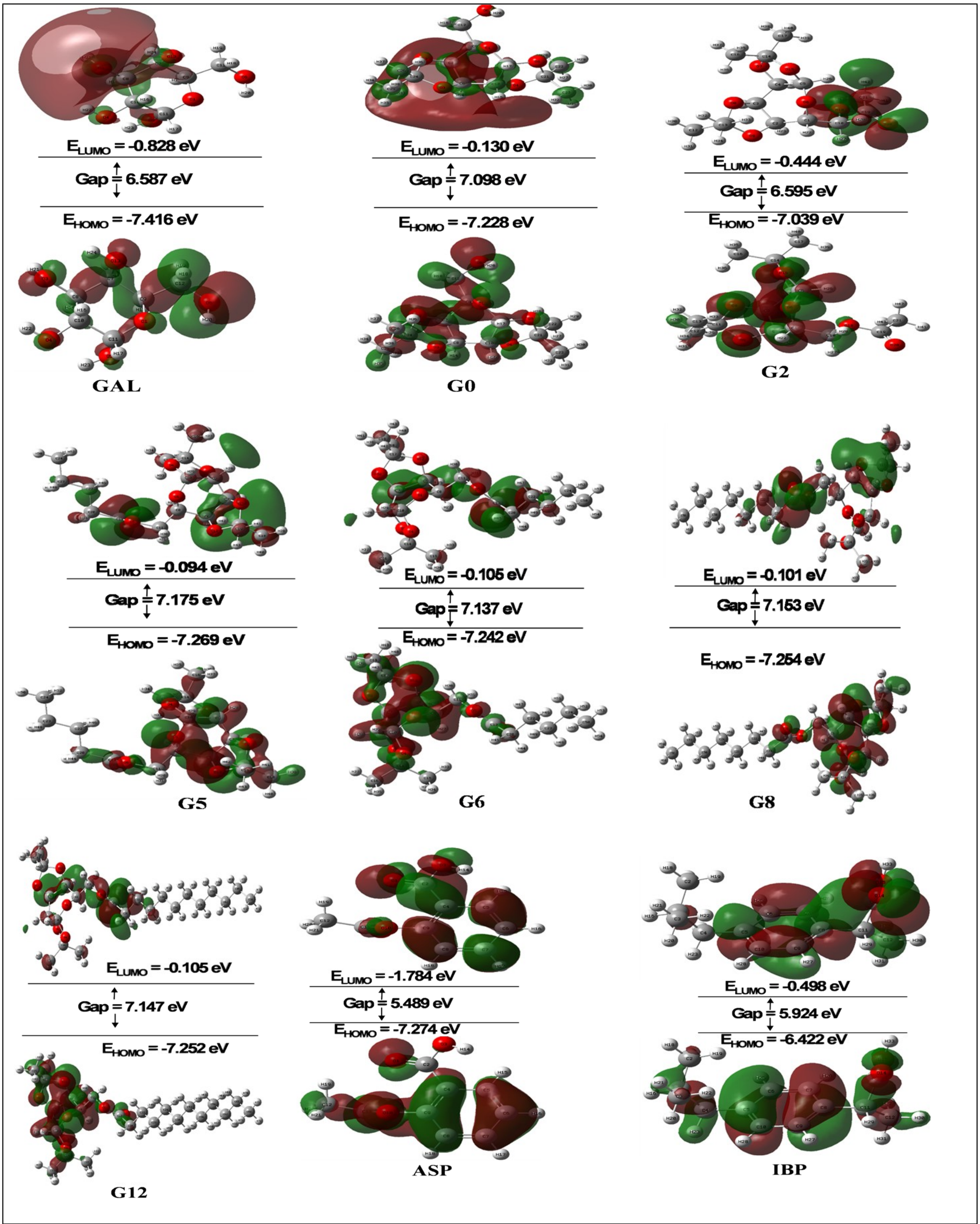

Figure S3. Frontier molecular orbital (HOMO and LUMO) and related energies of synthesized esters.

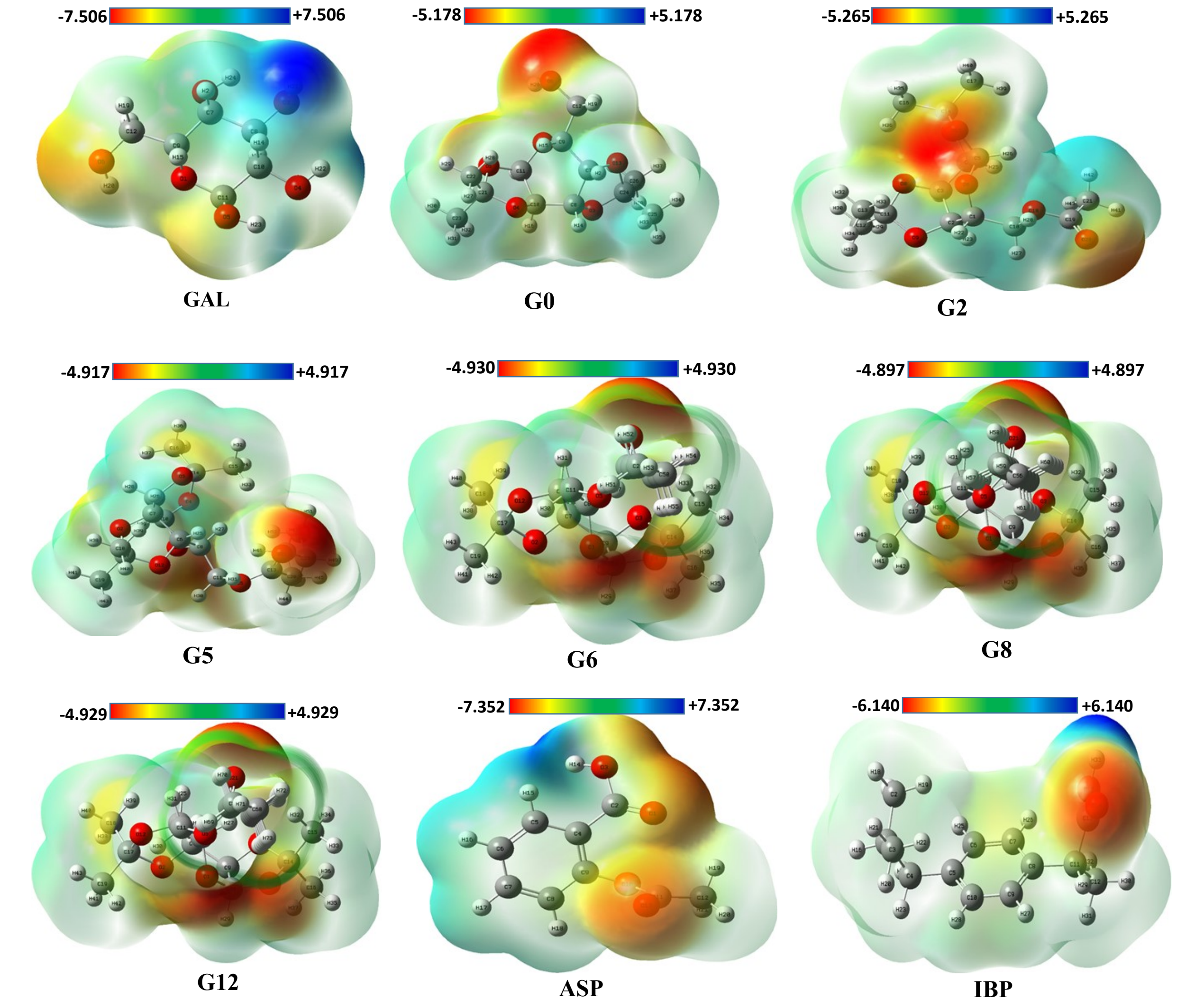

Figure S4. Molecular electrostatic potential map of SEs along with reference drugs.

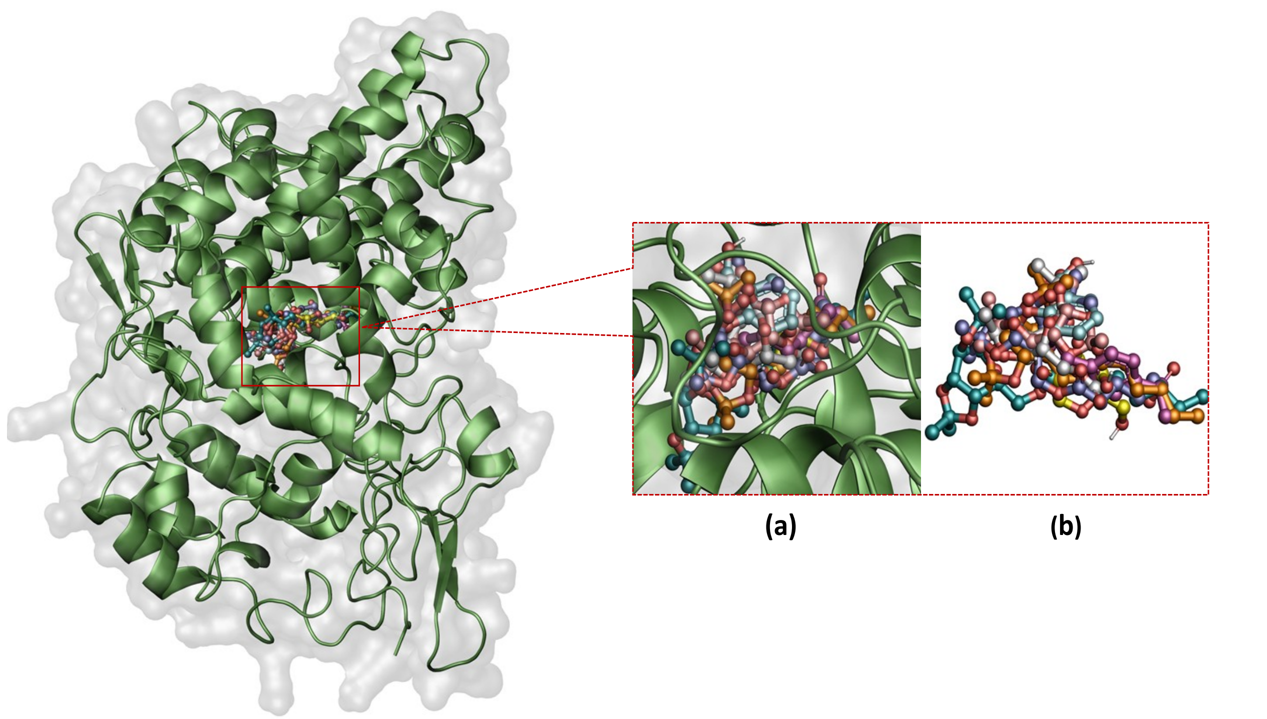

Figure S5. Docked conformer of all the ligands along with reference drug interacting in the same binding pocket of the receptor protein (PDB ID: 5F19).

.

Table S1. Energy (eV) of HOMO-LUMO, gap, hardness (*η*), softness (*S*), chemical potential (*μ*), electronegativity (*χ*), and electrophilicity (*ω*) of SEs and standard drugs.

Drug	eHOMO (H)	eLUMO (L)	Gap (ΔE)	Hardness (η)	Softness (S)	Chemical potential (μ)	Electronegativity (χ)	Global electrophilicity index (*ω*)
GAL	-7.416	-0.828	6.587	3.2935	0.3036	-4.122	4.1220	2.579457
G0	-7.228	-0.130	7.098	3.549	0.2818	-3.679	3.6790	1.906881
G2	-7.039	-0.444	6.595	3.2975	0.3033	-3.742	3.7415	2.122642
G5	-7.269	-0.094	7.175	3.5875	0.2787	-3.682	3.6815	1.888981
G6	-7.242	-0.105	7.137	3.5685	0.2802	-3.674	3.6735	1.890795
G8	-7.254	-0.101	7.153	3.5765	0.2796	-3.678	3.6775	1.890676
G12	-7.252	-0.105	7.147	3.5735	0.2798	-3.679	3.6785	1.893293
ASP	-7.274	-1.784	5.489	2.7445	0.3644	-4.529	4.5290	3.736899
IBP	-6.422	-0.498	5.924	2.9620	0.3376	-3.460	3.4600	2.020864

Table S2. Binding affinity, nonbonding interactions of Galactose derivatives, and the standard drugs with the receptor, COX-2 (5F19).

Name	Binding affinity (kcal/mol)	Residue in Contact	Bond distance (Å)	Interaction type	2D diagram
GAL	-6.7	LEU531	2.854	H	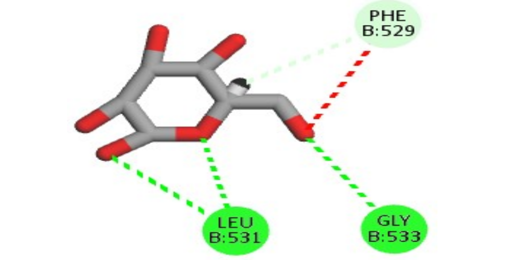
		LEU531	1.945	H	
		GLY533	2.641	H	
		GLY533	2.975	CH	
		PHE529	2.305	CH	
G0	-8.6	GLY526	2.974	CH	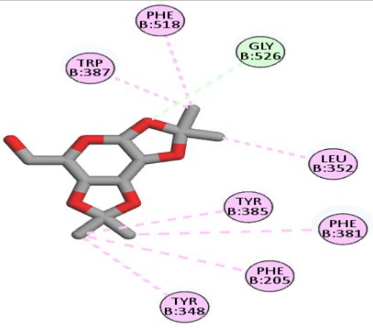
		LEU352	4.896	A	
		PHE205	5.109	PA	
		TYR348	4.458	PA	
		PHE381	4.806	PA	
		TYR385	5.302	PA	
		TRP387	5.060	PA	
		PHE518	4.336	PA	
G2	-9.7	GLY526	2.748	CH	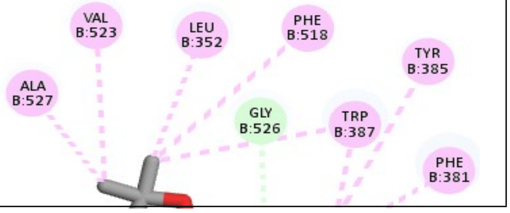
		ALA527	4.210	A	
		VAL523	3.953	A	
		LEU352	4.003	A	
		LEU384	5.096	A	

		LEU384	5.331	A	
		PHE381	3.775	PA	
		TYR385	5.038	PA	
		TRP387	4.757	PA	
		TRP387	4.890	PA	
		TRP387	4.648	PA	
		PHE518	4.235	PA	
G5	-9.7	ALA527	3.697	A	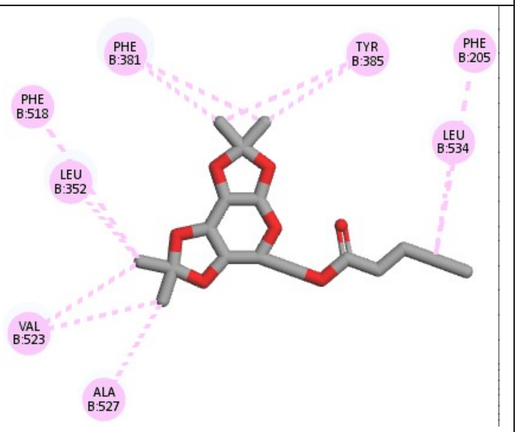
		LEU352	4.169	A	
		VAL523	4.353	A	
		VAL523	3.788	A	
		LEU534	4.163	A	
		PHE205	5.459	PA	
		PHE381	4.418	PA	
		PHE381	3.988	PA	
		TYR385	4.180	PA	
		TYR385	5.038	PA	
		PHE518	4.573	PA	
G6	-10.1	VAL523	2.702	CH	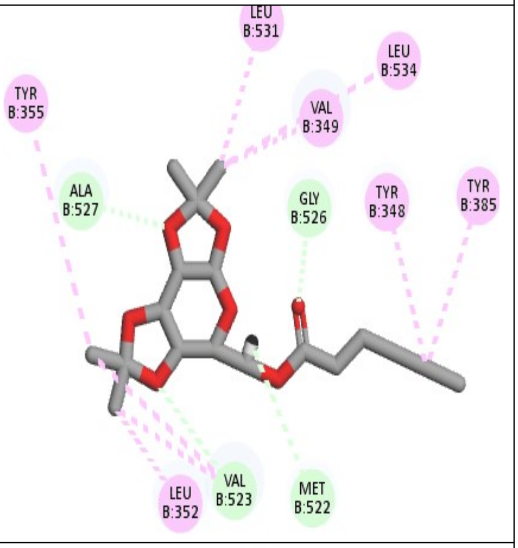
		GLY526	2.448	CH	
		ALA527	2.261	CH	
		MET522	2.633	CH	
		VAL349	3.757	A	
		LEU531	4.731	A	
		LEU534	5.025	A	
		VAL523	3.425	A	
		LEU352	3.965	A	
		VAL523	4.656	A	
		TYR348	5.061	PA	
		TYR355	5.487	PA	
		TYR385	4.656	PA	
G8	-10.4	VAL523	2.445	CH	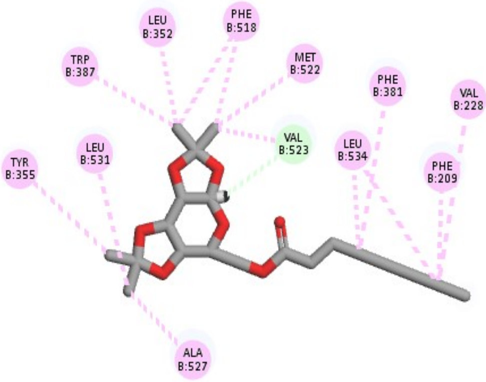
		ALA527	3.034	A	
		LEU352	4.079	A	
		MET522	5.265	A	
		VAL523	4.179	A	
		LEU531	5.425	A	
		LEU534	4.527	A	
		VAL228	4.931	A	
		LEU534	4.946	A	
		PHE209	4.487	PA	
		TYR355	4.221	PA	
		PHE381	5.141	PA	
		TRP387	4.308	PA	
		PHE518	4.442	PA	

		PHE518	3.582	PA	
G10	-8.8				
G12	-2.3				
ASP	-7.4	PHE381	2.661	H	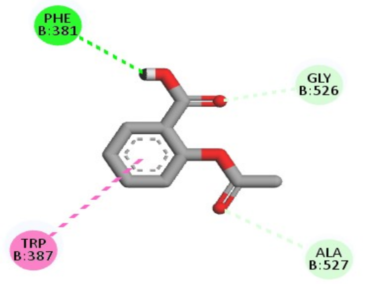
		GLY526	2.565	CH	
		ALA527	2.828	CH	
		TRP387	5.077	PPTsh	
IBP	-9	PHE529	2.158	H	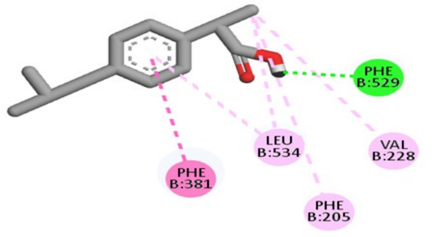
		PHE381	5.128	PPTsh	
		VAL228	4.877	A	
		LEU534	3.972	A	
		PHE205	5.148	PA	
		LEU534	5.235	PA	
Rxb	-9.4	MET522	1.930	CH	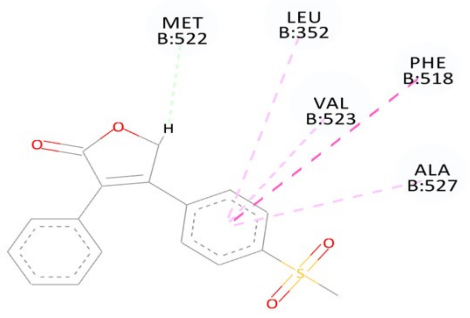
		PHE518	5.581	PPTsh	
		LEU352	5.281	PA	
		VAL523	4.574	PA	
		ALA527	4.898	PA	
		-	-	-	

H = Conventional hydrogen bond, CH = Carbon Hydrogen Bond, A = Alkyl, PA = Pi-Alkyl, PS = Pi-sigma, PPTSh =Pi-Pi T-shaped.

Field-based 3D-QSAR model for pKi prediction

A QSAR model quantitatively assesses the relationship between the molecular structures of a series of compounds and their corresponding biological activities. The 3D-QSAR method operates under the assumption that compounds exhibiting structural or physicochemical similarities are likely to demonstrate similar biological activities. A collection of 22 structurally diverse COX-1 inhibitors, with inhibition constant (Ki) values spanning from 7 to 9300 nM, was obtained from the BindingDB database[1-10] (Table S3). Additionally, 25 COX-2 inhibitors, exhibiting a broad spectrum of biological activity with Ki values ranging between 3 and 14,790 nM, were also compiled (Table S5). The structures of the molecules were processed using the LigPrep module of Maestro Version 12.5.139 with the following parameters: (i) the OPLS3e force field was applied (ii) the ionization state was neutral (iii) chiralities were determined from 3D structure and (iv) one low energy conformer per ligand per ligand was generated. Alignment of molecules is

the most essential input for creating a highly predictive field-based 3D-QSAR model.[1-10] Hence all the LigPrep output molecules were subjected to flexible ligand alignment. Alignment procedure was carried out separately for COX-1 and COX-2 inhibitors aiming to develop two field based 3D-QSAR model. During flexible ligand alignment reference structure was chosen automatically. Thorough sampling method was adopted with the maximum number of conformers of 1000 and nonbonded close contact distance of 0.5 Å.

Two field-based 3D-QSAR models were developed using aligned molecular structures. Both datasets demonstrate diversity in their pharmacological and structural properties. The Ki values (nM) were converted to pKi using the standard conversion formula: $pKi = -\log(10^{-9} \times Ki)$. Each dataset was randomly partitioned into a training set (70%) and a test set (30%), with a partial least squares (PLS) factor of 3 applied. The random selection performed by the software was subsequently verified through visual inspection to ensure that the training and test sets maintained diversity. In this study, interaction energy calculations for the 3D QSAR model were conducted utilizing five different fields such as steric, electrostatic, hydrophobic, hydrogen bond donor (HBD), and hydrogen bond acceptor (HBA). These calculations were performed using Gaussian equations to evaluate the respective fields.[8-12] The optimal model was chosen based on its statistical robustness. Validation was conducted using test set comprising 30% compounds of the both dataset. Key parameters for evaluating the test set included RMSE, Q^2, and Pearson's r (Table S7, S9), which collectively reflect the model's predictive accuracy. To prevent overfitting, the number of PLS factors was carefully limited to 3. Additionally, scatter plots were generated to illustrate the correlation between the observed and predicted activities of the dataset molecules (Figure S6).

Table S3 Dataset-1 for QSAR model of COX-1 inhibition

BindingDB MonomerID	IUPAC name	Ki (nM)	pKi	Reference
11639	4-(5-(p-tolyl)-3-(trifluoromethyl)-1H-pyrazol-1-yl)benzenesulfonamide	1200	5.921	[1]
13063	4-(5-methyl-3-phenylisoxazol-4-yl)benzenesulfonamide	250	6.602	[2]
22369	4-(4-(methylsulfonyl)phenyl)-3-phenylfuran-2(5H)-one	5300	5.276	[3]
50295287	2-(1-methyl-5-(4-methylbenzoyl)-1H-pyrrol-2-yl)acetic acid	1080	5.967	[4]
50027952	2-(5-(4-chlorobenzoyl)-1,4-dimethyl-1H-pyrrol-2-yl)acetic acid	430	6.367	[1]
50090676	2-(4-(thiophene-2-carbonyl)phenyl)propanoic acid	1100	5.959	[1]
50074922	2-(2-fluoro-[1,1'-biphenyl]-4-yl)propanoic acid	410	6.387	[5]
85514	2-((3-(trifluoromethyl)phenyl)amino)benzoate	3000	5.523	[1]
50009859	2-(4-isobutylphenyl)propanoic acid	4800	5.319	[6]
17638	2-(1-(4-chlorobenzoyl)-5-methoxy-2-methyl-1H-indol-3-yl)acetic acid	190	6.721	[7]
50022271	2-(3-benzoylphenyl)propanoic acid	47	7.328	[1]
85512	4-isopropyl-2-(4-methoxyphenyl)-1H-naphtho[1,2-d]imidazole	7600	5.119	[1]
50056998	(Z)-4-hydroxy-2-methyl-N-(5-methylthiazol-2-yl)-2H-benzo[e][1,2]thiazine-3-carbimidic acid 1,1-dioxide	5700	5.244	[1]
50056999	N-(4-nitro-2-phenoxyphenyl)methanesulfonamide	4100	5.387	[6]
22360	2-acetoxybenzoic acid	4450	5.352	8
50009860	2-(6-methoxynaphthalen-2-yl)propanoic acid	9300	5.032	1
50331888	5-chloro-2-hydroxy-3-(thiophene-2-carbonyl)-1H-indole-1-carbimidic acid	81	7.092	1
50134036	2-((2,3-dimethylphenyl)amino)benzoic acid	1940	5.712	8
54705	2-(3-phenoxyphenyl)propanoic acid	2730	5.564	8
50097346	2-(6-chloro-9H-carbazol-2-yl)propanoic acid	87	7.060	1

50174201	2-(2-((2,6-dichlorophenyl)amino)phenyl)acetate	7	8.155	6
50016799	2-(1,8-diethyl-1,3,4,9-tetrahydropyrano[3,4-b]indol-1-yl)acetic acid	1200	5.921	6

Table S4 QSAR study of Dataset-1

Ligand Name	QSAR Set	Activity (pKi)	PLS Factors	Predicted Activity	Prediction Error
11639	test	5.921	1	6.68048	0.759661
			2	6.56598	0.645162
			3	6.75655	0.835734
13063	training	6.602	1	6.72904	0.12698
			2	6.66233	0.060271
			3	6.86074	0.258685
22369	test	5.276	1	6.35229	1.07657
			2	6.42641	1.15069
			3	6.65428	1.37855
50295287	training	5.967	1	6.27645	0.309873
			2	6.49692	0.530347
			3	6.21237	0.245799
50027952	test	6.367	1	6.37389	0.00736
			2	6.66216	0.295633
			3	6.39338	0.026845
50090676	training	5.959	1	6.17413	0.215522
			2	5.93978	-0.01882
			3	5.67707	-0.28154
50074922	training	6.387	1	5.95501	-0.43221
			2	5.8935	-0.49372
			3	5.98445	-0.40277
85514	test	5.523	1	6.22665	0.703771
			2	5.72719	0.204312
			3	5.74082	0.217946
50009859	training	5.319	1	5.91738	0.59862

			2	5.63558	0.316821
			3	5.70458	0.38582
17638	training	6.721	1	6.36328	-0.35797
			2	6.7427	0.021449
			3	6.45498	-0.26626
50022271	training	7.328	1	6.63582	-0.69208
			2	6.62631	-0.70159
			3	6.8851	-0.4428
85512	training	5.119	1	5.80526	0.686076
			2	5.01711	-0.10208
			3	4.90771	-0.21147
50056998	training	5.244	1	4.36267	-0.88146
			2	5.15413	-0.08999
			3	5.32789	0.083767
50056999	training	5.387	1	6.01241	0.625195
			2	5.51257	0.125353
			3	5.52255	0.135336
22360	training	5.352	1	5.3854	0.033756
			2	5.36121	0.009569
			3	5.36694	0.015298
50009860	test	5.032	1	5.59785	0.566328
			2	5.17746	0.145943
			3	4.9973	-0.03422
50331888	training	7.092	1	6.77444	-0.31708
			2	7.58131	0.489791
			3	7.30359	0.212074
50134036	training	5.712	1	6.30049	0.588292
			2	5.85864	0.146438
			3	5.90568	0.193479
54705	training	5.564	1	6.3169	0.753063
			2	5.73069	0.16685
			3	5.78964	0.225805

50097346	test	7.06	1	6.26494	-0.79554
			2	6.44114	-0.61934
			3	6.44349	-0.61699
50174201	training	8.155	1	7.13596	-1.01894
			2	7.79004	-0.36486
			3	8.11798	-0.03693
50016799	training	5.921	1	5.68317	-0.23765
			2	5.82499	-0.09582
			3	5.80653	-0.11429

Table S5 Dataset-2 for QSAR model of COX-2 inhibition

BindingDB MonomerID	IUPAC name	Ki (nM)	pKi	References
50056999	N-(4-itro-2-phenoxyphenyl)methanesulfonamide	560	6.251812	6
50029593	N-(2-(cyclohexyloxy)-4-nitrophenyl)methanesulfonamide	350	6.455932	1
11639	4-(5-(p-tolyl)-3-(trifluoromethyl)-1H-pyrazol-1-yl)benzenesulfonamide	830	6.080922	1
50029616	5-(4-fluorophenyl)-1-(4-(methylsulfonyl)phenyl)-3-(trifluoromethyl)-1H-pyrazole	2000	5.69897	1
50072064	5-chloro-6'-methyl-3-(4-(methylsulfonyl)phenyl)-2,3'-bipyridine	69	7.161151	9
13063	4-(5-methyl-3-phenylisoxazol-4-yl)benzenesulfonamide	870	6.060481	6
50285227	2-(4-fluorophenyl)-3-(4-(methylsulfonyl)phenyl)thiophene	250	6.60206	10
50566911	1-((5-(hydroxymethyl)-3-(4-(methylsulfonyl)phenyl)-6-(3,3,3-trifluoropropoxy)pyridin-2-yl)oxy)-2-methylpropan-2-ol	32	7.49485	9
50566912	1-((5-(hydroxymethyl)-3-(4-(methylsulfonyl)phenyl)-6-(4,4,4-trifluorobutoxy)pyridin-2-yl)oxy)-2-methylpropan-2-ol	12	7.920819	9
50566900	1-((5-(hydroxymethyl)-3-(4-(methylsulfonyl)phenyl)-6-propoxypyridin-2-yl)oxy)-2-methylpropan-2-ol	114	6.943095	9
50566901	1-((6-butoxy-5-(hydroxymethyl)-3-(4-(methylsulfonyl)phenyl)pyridin-2-yl)oxy)-2-methylpropan-2-ol	12	7.920819	9
50566910	1-(((6-butoxy-5-(hydroxymethyl)-3-(4-(methylsulfonyl)phenyl)pyridin-2-	3	8.522879	9

	yl)oxy)methyl)cyclobutan-1-ol			
22369	4-(4-(methylsulfonyl)phenyl)-3-phenylfuran-2(5H)-one	530	6.275724	6
50566913	6'-butoxy-2'-(2-hydroxy-2-methylpropoxy)-5'-(hydroxymethyl)-[2,3'-bipyridine]-5-sulfonamide	19	7.721246	9
50114414	2-((5-(4-aminophenyl)-1,3,4-oxadiazol-2-yl)methyl)-5,6-diphenyl-1,2,4-triazin-3(2H)-one	14620	4.835053	10
50114416	2-((5-(4-methoxyphenyl)-1,3,4-oxadiazol-2-yl)methyl)-5,6-diphenyl-1,2,4-triazin-3(2H)-one	14750	4.831208	10
50114415	2-((5-(4-chlorophenyl)-1,3,4-oxadiazol-2-yl)methyl)-5,6-diphenyl-1,2,4-triazin-3(2H)-one	14710	4.832387	10
50114417	2-((5-(4-nitrophenyl)-1,3,4-oxadiazol-2-yl)methyl)-5,6-diphenyl-1,2,4-triazin-3(2H)-one	14790	4.830032	10
198171	2-((5-((4-methoxyphenyl)amino)-1,3,4-oxadiazol-2-yl)methyl)-5,6-diphenyl-1,2,4-triazin-3(2H)-one	650	6.187087	11
198174	2-((5-((4-methoxyphenyl)amino)-1,3,4-thiadiazol-2-yl)methyl)-5,6-diphenyl-1,2,4-triazin-3(2H)-one	770	6.113509	11
198172	2-((5-((4-chlorophenyl)amino)-1,3,4-oxadiazol-2-yl)methyl)-5,6-diphenyl-1,2,4-triazin-3(2H)-one	630	6.200659	11
198173	2-((5-((4-nitrophenyl)amino)-1,3,4-oxadiazol-2-yl)methyl)-5,6-diphenyl-1,2,4-triazin-3(2H)-one	500	6.30103	11
198175	2-((5-((4-chlorophenyl)amino)-1,3,4-thiadiazol-2-yl)methyl)-5,6-diphenyl-1,2,4-triazin-3(2H)-one	960	6.017729	11
198176	2-((5-((4-nitrophenyl)amino)-1,3,4-thiadiazol-2-yl)methyl)-5,6-diphenyl-1,2,4-triazin-3(2H)-one	880	6.055517	11
50114413	2-((5-(2,4-dihydroxyphenyl)-1,3,4-oxadiazol-2-yl)methyl)-5,6-diphenyl-1,2,4-triazin-3(2H)-one	14510	4.838333	10

Table S6 QSAR study of Dataset-2

Ligand Name	QSAR Set	Activity (pKi)	# Factors	Predicted Activity	Prediction Error
50056999	test	6.252	1	5.94542	-0.3064
			2	6.16716	-0.08465
			3	6.5233	0.271486
50029593	test	6.456	1	5.9997	-0.45623
			2	6.22924	-0.22669
			3	6.59617	0.140234

11639	training	6.081	1	5.77837	-0.30255
			2	6.43978	0.358859
			3	6.19944	0.118521
50029616	test	5.699	1	5.83917	0.140202
			2	6.49389	0.794919
			3	6.35792	0.658954
50072064	training	7.161	1	6.99964	-0.16151
			2	6.89043	-0.27072
			3	6.81494	-0.34621
13063	training	6.06	1	5.81044	-0.25004
			2	6.45076	0.390277
			3	6.15582	0.095335
50285227	training	6.602	1	5.8279	-0.77416
			2	6.49511	-0.10695
			3	6.3565	-0.24556
50566911	test	7.495	1	7.65283	0.157978
			2	7.63803	0.143175
			3	7.6332	0.138347
50566912	training	7.921	1	7.64435	-0.27647
			2	7.64607	-0.27474
			3	7.68693	-0.23388
50566900	training	6.943	1	7.67167	0.728576
			2	7.6392	0.696103
			3	7.60253	0.65943
50566901	training	7.921	1	7.65607	-0.26475
			2	7.6472	-0.27362
			3	7.67321	-0.24761
50566910	test	8.523	1	7.63829	-0.88459
			2	7.60529	-0.91759
			3	7.62547	-0.89741
22369	training	6.276	1	6.80279	0.527069
			2	6.60042	0.324698

			3	6.33975	0.064024
50566913	training	7.721	1	7.66388	-0.05736
			2	7.67995	-0.0413
			3	7.7181	-0.00314
50114414	training	4.835	1	5.53751	0.702453
			2	5.16416	0.329102
			3	4.75584	-0.07922
50114416	training	4.832	1	5.60712	0.774731
			2	5.33018	0.497792
			3	5.0898	0.257416
50114415	training	4.832	1	5.60712	0.774731
			2	5.33018	0.497792
			3	5.0898	0.257416
50114417	training	4.83	1	5.58482	0.754792
			2	5.27574	0.445705
			3	4.97585	0.145822
198171	training	6.187	1	5.73182	-0.45527
			2	5.66877	-0.51831
			3	6.13505	-0.05204
198174	test	6.114	1	5.7765	-0.33701
			2	5.76725	-0.34626
			3	6.30364	0.190131
198172	training	6.201	1	5.71884	-0.48182
			2	5.63359	-0.56707
			3	6.03264	-0.16802
198173	training	6.301	1	5.72824	-0.57279
			2	5.66365	-0.63738
			3	6.12918	-0.17185
198175	training	6.018	1	5.76367	-0.25406
			2	5.73094	-0.28678
			3	6.19627	0.17854
198176	training	6.056	1	5.77364	-0.28188

			2	5.76099	-0.29453
			3	6.2909	0.235383
50114413	training	4.838	1	5.48337	0.645034
			2	5.0672	0.228865
			3	4.63139	-0.20694

Table S7 Statistical parameters field-based QSAR model for COX-1 inhibition

# PLS Factors	SD	R^2	R^2 CV	R^2 Scramble	Stability	F	P	RMSE	Q^2	Pearson-r
1	0.6011	0.5528	0.3136	0.418	0.175	17.3	0.000963	0.73	0.5476	0.4423
2	0.3495	0.8596	0.3277	0.5307	0.243	39.8	2.87E-06	0.62	0.4584	0.6426
3	0.2891	0.9114	0.4975	0.7225	0.233	41.1	1.37E-06	0.71	0.4669	0.5168

R^2 = correlation coefficient of experimentally observed and predicted activity of the training set; SD = standard deviation of regression; R^2 CV=cross-validated value (leave-one-out validation); F = variance ratio; p = statistical significance; Q^2 = value of R^2 of the test set; RMSE = root mean square error; Pearson's r = correlation co efficient of predicted and experimentally observed activity of the test set.

Table S8 Contribution factors of field-based QSAR model for COX-1 inhibition

# PLS Factors	Gaussian Steric	Gaussian Electrostatic	Gaussian Hydrophobic	Gaussian Hbond Acceptor	Gaussian Hbond Donor
1	0.271	0.089	0.238	0.218	0.183
2	0.256	0.103	0.206	0.233	0.202
3	0.251	0.104	0.208	0.236	0.201

Table S9 Statistical parameters of field-based QSAR model for COX-2 inhibition

# PLS Factors	SD	R^2	R^2 CV	R^2 Scramble	Stability	F	P	RMSE	Q^2	Pearson-r
1	0.5436	0.7277	0.6031	0.211	0.978	42.8	6.83E-06	0.51	0.7941	0.9153
2	0.4382	0.8341	0.6928	0.405	0.966	37.7	1.41E-06	0.51	0.7876	0.9027
3	0.2729	0.9400	0.8404	0.5672	0.958	73.1	8.61E-09	0.45	0.8398	0.9357

R^2 = correlation coefficient of experimentally observed and predicted activity of the training set; SD = standard deviation of regression; R^2 CV=cross-validated value (leave-one-out validation); F = variance ratio; p = statistical significance; Q^2 = value of R^2 of the test set; RMSE = root mean square error; Pearson's r = correlation co efficient of predicted and experimentally observed activity of the test set.

Table S10 Contribution factors of field-based QSAR model for COX-2 inhibition

# Factors	Gaussian Steric	Gaussian Electrostatic	Gaussian Hydrophobic	Gaussian Hbond Acceptor	Gaussian Hbond Donor
1	0.31	0.116	0.175	0.291	0.108
2	0.317	0.116	0.178	0.277	0.112

3	0.346	0.104	0.184	0.225	0.142

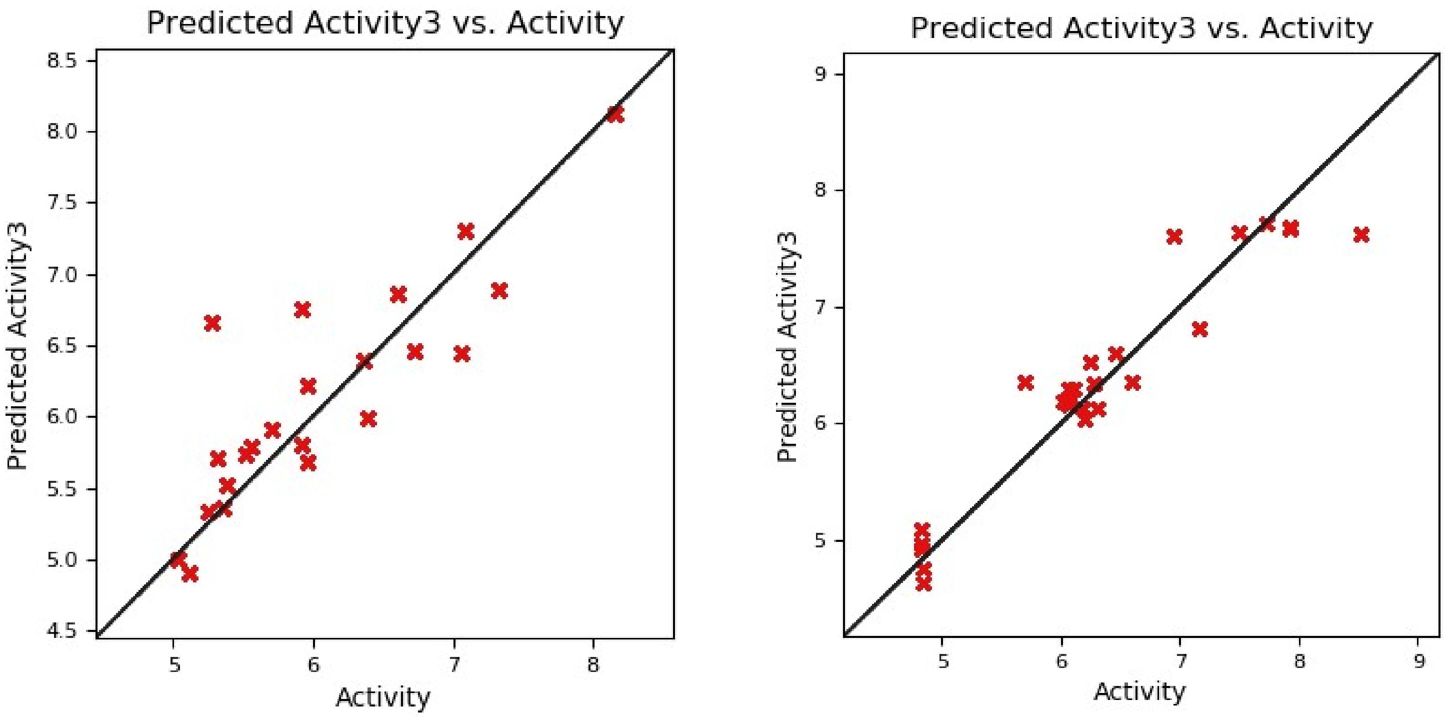

(a) (b)

Figure S6: Scatter plot for the comparison of actual versus predicted activity using the field-based QSAR model (PLS 3). The scatter plots depict the results for (a) the training and test set in the context of COX-1 inhibition, alongside (b) the training and test set for COX-2 inhibition.

References

[1] T. D. Warner, F. Giuliano, I. Vojnovic, A. Bukasa, J. A. Mitchell, and J. R. Vane, 'Nonsteroid drug selectivities for cyclo-oxygenase-1 rather than cyclo-oxygenase-2 are associated with human gastrointestinal toxicity: a full in vitro analysis.', Proc Natl Acad Sci U S A, vol. 96, no. 13, pp. 7563–8, Jun. 1999, doi: 10.1073/pnas.96.13.7563.

[2] D. J. Cochrane, B. Jarvis, and G. M. Keating, 'Etoricoxib.', Drugs, vol. 62, no. 18, pp. 2637–51; discussion 2652-3, 2002, doi: 10.2165/00003495-200262180-00006.

[3] B. Cryer and M. Feldman, 'Cyclooxygenase-1 and cyclooxygenase-2 selectivity of widely used nonsteroidal anti-inflammatory drugs.', Am J Med, vol. 104, no. 5, pp. 413–21, May 1998, doi: 10.1016/s0002-9343(98)00091-6.

[4] M. R. Weir, R. S. Sperling, A. Reicin, and B. J. Gertz, 'Selective COX-2 inhibition and cardiovascular effects: a review of the rofecoxib development program.', Am Heart J, vol. 146, no. 4, pp. 591–604, Oct. 2003, doi: 10.1016/S0002-8703(03)00398-3.

[5] B. Cryer and M. Feldman, 'Cyclooxygenase-1 and cyclooxygenase-2 selectivity of widely used nonsteroidal anti-inflammatory drugs.', Am J Med, vol. 104, no. 5, pp. 413–21, May 1998, doi: 10.1016/s0002-9343(98)00091-6.

[6] D. Riendeau et al., ‘Etoricoxib (MK-0663): preclinical profile and comparison with other agents that selectively inhibit cyclooxygenase-2.’, J Pharmacol Exp Ther, vol. 296, no. 2, pp. 558–66, Feb. 2001, [Online]. Available: http://www.ncbi.nlm.nih.gov/pubmed/11160644

[7] C. C. Chan et al., ‘Rofecoxib [Vioxx, MK-0966; 4-(4’-methylsulfonylphenyl)-3-phenyl-2-(5H)-furanone]: a potent and orally active cyclooxygenase-2 inhibitor. Pharmacological and biochemical profiles.’, J Pharmacol Exp Ther, vol. 290, no. 2, pp. 551–60, Aug. 1999, [Online]. Available: http://www.ncbi.nlm.nih.gov/pubmed/10411562

[8] B. Cryer and M. Feldman, ‘Cyclooxygenase-1 and cyclooxygenase-2 selectivity of widely used nonsteroidal anti-inflammatory drugs.’, Am J Med, vol. 104, no. 5, pp. 413–21, May 1998, doi: 10.1016/s0002-9343(98)00091-6.

[9] Z. Zhang et al., ‘Gut-Restricted Selective Cyclooxygenase-2 (COX-2) Inhibitors for Chemoprevention of Colorectal Cancer.’, J Med Chem, vol. 64, no. 15, pp. 11570–11596, Aug. 2021, doi: 10.1021/acs.jmedchem.1c00890.

[10] Z. Chen et al., ‘The Repertoire of Small-Molecule PET Probes for Neuroinflammation Imaging: Challenges and Opportunities beyond TSPO.’, J Med Chem, vol. 64, no. 24, pp. 17656–17689, Dec. 2021, doi: 10.1021/acs.jmedchem.1c01571.

[11] A. G. Banerjee, N. Das, S. A. Shengule, R. S. Srivastava, and S. K. Shrivastava, ‘Synthesis, characterization, evaluation and molecular dynamics studies of 5, 6-diphenyl-1,2,4-triazin-3(2H)-one derivatives bearing 5-substituted 1,3,4-oxadiazole as potential anti-inflammatory and analgesic agents.’, Eur J Med Chem, vol. 101, pp. 81–95, Aug. 2015, doi: 10.1016/j.ejmech.2015.06.020.

[12] A. G. Banerjee, N. Das, S. A. Shengule, P. A. Sharma, R. S. Srivastava, and S. K. Shrivastava, ‘Design, synthesis, evaluation and molecular modelling studies of some novel 5,6-diphenyl-1,2,4-triazin-3(2H)-ones bearing five-member heterocyclic moieties as potential COX-2 inhibitors: A hybrid pharmacophore approach.’, Bioorg Chem, vol. 69, pp. 102–120, Dec. 2016, doi: 10.1016/j.bioorg.2016.10.003.
